# PARP inhibitors restore NK cell function via secretory crosstalk with tumor cells in prostate cancer

**DOI:** 10.1172/JCI197157

**Published:** 2026-01-27

**Authors:** Zheng Chao, Le Li, Xiaodong Hao, Hao Peng, Yanan Wang, Chunyu Zhang, Xiangdong Guo, Peikun Liu, Sheng Ma, Junbiao Zhang, Guanyu Qu, Yuzheng Peng, Zhengping Wei, Jing Luo, Bo Liu, Peixiang Lan, Zhihua Wang

**Affiliations:** 1 Department of Urology, Tongji Hospital, Tongji Medical College, Huazhong University of Science and Technology, Wuhan, China.; 2 Institute of Organ Transplantation, Tongji Hospital, Tongji Medical College, Huazhong University of Science and Technology, Key Laboratory of Organ Transplantation, Ministry of Education, NHC Key Laboratory of Organ Transplantation, Key Laboratory of Organ Transplantation, Chinese Academy of Medical Sciences, Wuhan, China.; 3 Institute of Reproductive Health, Center for Reproductive Medicine and; 4 Department of Oncology, Tongji Hospital, Tongji Medical College, Huazhong University of Science and Technology, Wuhan, China.; 5 Taikang Tongji (Wuhan) Hospital, Wuhan, China.

**Keywords:** Immunology, Oncology, Cancer immunotherapy, NK cells, Prostate cancer

## Abstract

Prostate cancer (PCa) is one of the most frequently diagnosed malignancies and the main cause of cancer-related death in men worldwide. Poly(ADP-ribose) polymerase inhibitors (PARPi) have been approved for the treatment of PCa harboring *BRCA1/2* mutations. While the survival benefits conferred by PARPi may extend beyond this specific patient population based on evidence from recent clinical trials, the underlying mechanisms remain unexplored. Here, we demonstrate that PARPi substantially restored NK cell functions by promoting cyclophilin A (CypA) secretion from PCa cells, which correlated with improved prognosis in PCa patients from our and public cohorts. Mechanistically, tumor-derived CypA specifically from PCa cells bound to ANXA6 and activated the downstream FPR1 signaling pathway, leading to increased mitochondrial oxidative phosphorylation and NK cell activation. Pharmacological inhibition of CypA blocked FPR1/AKT signaling and diminished the cytotoxic effects of NK cells, thereby compromising the therapeutic efficacy of PARPi against PCa. Conversely, combining NK cell adoptive transfer therapy with PARPi markedly prolonged survival in mice bearing PCa. Collectively, we reveal a unique secretory crosstalk between PCa cells and NK cells induced by PARPi and propose a promising strategy for treating PCa.

## Abstract 2

PARPi induces a novel immune crosstalk mechanism in prostate cancer and restore NK cells antitumor effect.

## Graphical abstract

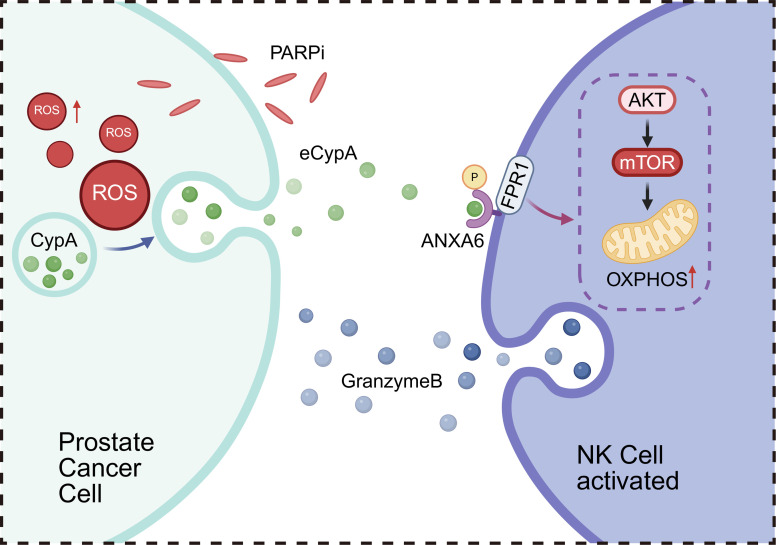

## Introduction

A significant proportion of patients with prostate cancer (PCa) progress to castration-resistant prostate cancer (CRPC) following androgen deprivation therapy (1). Among metastatic CRPC cases, homologous recombination repair (HRR) gene mutations are detected in 25%–30% of patients (2, 3), with *BRCA1/2* alterations being associated with highly aggressive PCa (4). This has led to the widespread application of poly(ADP-ribose) polymerase inhibitors (PARPi) in the treatment of PCa patients with homologous recombination deficiency (5). However, recent findings from multiple clinical studies suggest that the potential beneficiary population of PARPi may extend beyond this subset and could be associated with the activation of immune responses (5–8).

PCa is characterized as a typical cold tumor in the immunological context, primarily due to its limited neoantigen and downregulated HLA-I expression (9). This inherent feature results in the failure of therapies that typically activate CD8^+^ T cell–specific antitumor immunity in PCa (10). Concurrently, NK cells, which possess the unique capability to kill tumor cells independently of tumor neoantigens, have increasingly emerged as a promising direction in the development of alternative therapeutic strategies for PCa (11, 12). Previous studies demonstrate that Olaparib, a PARPi, substantially enhances NK cell–mediated cytotoxicity and antibody-dependent cellular cytotoxicity against both *BRCA* WT and mutant PCa cells (13), highlighting the crucial role of NK cells in PARPi-induced antitumor response (14). The MHC-unrestricted tumor recognition mechanism of NK cells (15) bypasses immune evasion caused by frequent MHC downregulation in PCa (16, 17), suggesting promising potential for autologous or allogeneic NK cell therapies and alternative strategies to enhance antitumor immune response in immune-cold tumors such as PCa (11).

Current preclinical and clinical strategies for NK cell–based therapies primarily involve direct stimulation and adoptive transfer approaches (18). However, the existence of complex crosstalk between NK cells and tumor cells can substantially influence the ultimate efficacy of NK cell therapies. For instance, in the hepatocellular carcinoma microenvironment, dysregulated serine metabolism restricts NK cell membrane protrusion formation, thereby impairing their ability to kill tumor cells (19). In PCa, the reactivation of retinoic acid receptors triggers a robust tumor senescence response and strongly enhances NK-mediated tumor clearance in a NKG2D-dependent manner (11). Thus, further exploration of specific activation targets for NK cells in PCa and the detailed mechanisms of crosstalk between NK cells and cancer cells will undoubtedly provide deeper insights into the treatment of PCa.

Here, we found that PARPi markedly induce the secretion of extracellular proteins by PCa cells, among which CypA is identified as a key mediator of NK cell activation. We reveal that PARPi induce CypA secretion from PCa cells, subsequently reprogramming mitochondrial metabolism in NK cells. This tumor-immune crosstalk mechanism overcomes 2 major barriers of NK cell immunotherapy — limited tumor infiltration and microenvironmental suppression — suggesting a promising PARPi-based combination strategy for CRPC treatment in a “like cures like” way.

## Results

### PARPi restore NK cell functions independent of BRCA status in PCa.

**Figure 1:**
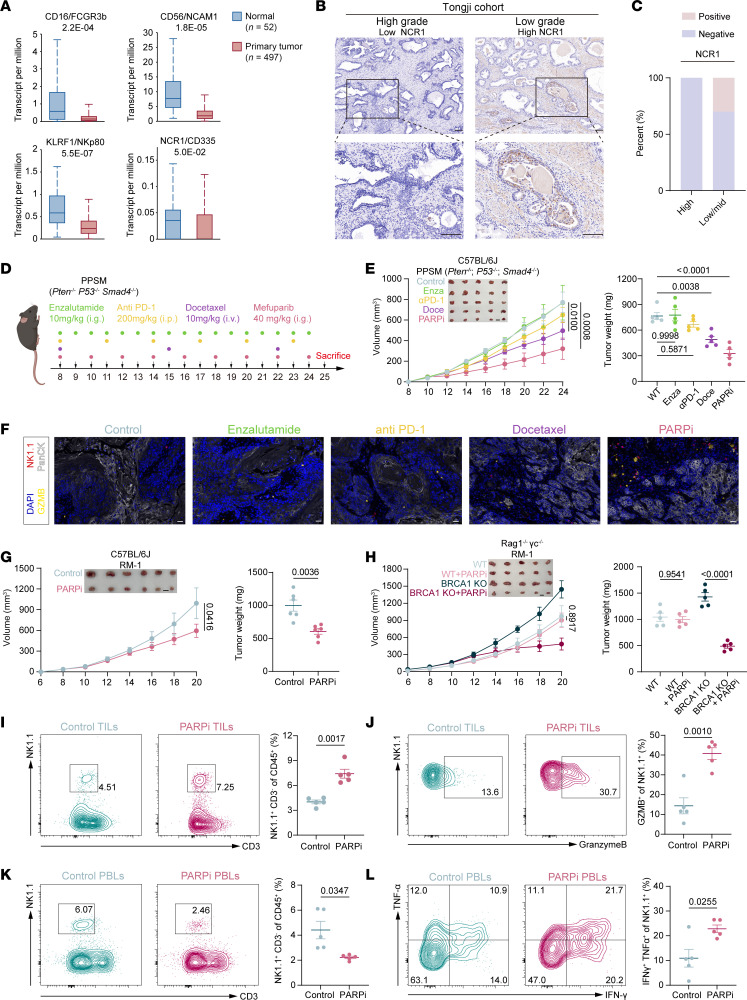
PARPi revitalize NK cells in the PCa microenvironment. (**A**) Expression of CD16, CD56, NCR1, and KLRF1 in PCa versus normal tissues (TCGA). The box-and-whisker plots depict the minimum and maximum values (whiskers), the upper and lower quartiles, and the median. (**B**) Immunohistochemical staining of NCR1 in high- and low-grade PCa tissues collected at Tongji Hospital. Scale bars: 100 μm. (**C**) Proportion of NCR1 expression in patients with high-grade (*N* = 10) versus intermediate/low-grade (*N* = 10) PCa. (**D**) Treatment regimens for C57BL/6 mice bearing CRPC-PPSM starting on day 8 after inoculation: enzalutamide (10 mg/kg, oral, daily), anti–PD-1 (200 μg/kg, i.p., every 3 days), docetaxel (10 mg/kg, i.v., weekly), and mefuparib (40 mg/kg, oral, every 2 days). Mice were euthanized on day 25 after tumor inoculation. (**E**) Tumor growth curves and tumor weights in mice from **D** for each treatment; *N* = 5 per group. Scale bars: 1 cm. (**F**) Immunofluorescence of tumors from **D** showing DAPI (blue), NK1.1 (red), GZMB (yellow), and PanCK (white). Scale bars: 20 μm. (**G**) Tumor growth curves and weights of C57BL/6 mice inoculated with RM-1 cells and treated with or without PARPi; *N* = 6 per group. Scale bars: 1 cm. (**H**) Tumor growth curves and weights of *Rag1*^–*/*–^ *γc*^–*/*–^ mice inoculated with RM-1 or RM-1 *BRCA1*-KO cells and treated with or without PARPi; *N* = 5 per group. Scale bars: 1 cm.(**I**–**L**) Flow cytometry analysis of NK cell proportions in tumors (**I**) and peripheral blood (**K**), GZMB^+^ proportion in TINKs (**J**), and TNF-α^+^ IFN-γ^+^ proportions in PBNKs (**L**) of mice from **G**; *N* = 5 per group. Tumor growth curve data are presented as mean ± SD and were analyzed by 2-way ANOVA with Tukey’s multiple-comparison test. Other data are presented as mean ± SEM. Data were analyzed by 1-way ANOVA (**E** and **H**) and Welch’s *t* test (**G** and **I**–**L**).

As NK cells play vital roles in PCa development, we first analyzed genes related to NK cell characteristics in The Cancer Genome Atlas (TCGA) PCa database. Levels of CD16 and CD56, as surface markers of human NK cells, and natural cytotoxicity triggering receptor 1 (NCR1) and killer cell lectin like receptor F1 (KLRF1), which characterize the cytotoxic effects of NK cells (20), were all clearly lower in PCa tissues, indicating a dual inhibition of the number and functions of NK cells in the PCa microenvironment (Figure 1A and Supplemental Figure 1A; supplemental material available online with this article; https://doi.org/10.1172/JCI197157DS1). Subgroups of patients with PCa and high expression of these 4 genes characterizing NK cell features tended to have a better prognosis (Supplemental Figure 1B). At the same time, we performed immunohistochemistry on PCa tissue collected from Tongji Hospital, using NCR1 to measure tumor-infiltrating NK cells (TINKs), and observed uniformly negative results in high-grade PCa (Figure 1, B and C). High-grade PCa exhibits metabolic characteristics characterized by high expression of lactate dehydrogenase A and elevated lactylation levels (Supplemental Figure 1, C and D), shaping an immunosuppressive tumor microenvironment (TME) (21–23), and is associated with poorer survival prognosis and impaired NK cell functions (Supplemental Figure 1, E and F) (24). Therefore, we attempted to find treatment regimens to revitalize TINKs. We used PPSM (*Pten*^–/–^ *P53*^–/–^ *Smad4*^–/–^) to mimic CRPC (25) and treated tumor-bearing mice with existing mainstream clinical treatment regimens for PCa (Figure 1D) and found that PARPi had the best therapeutic effect (Figure 1E and Supplemental Figure 2A). After immunofluorescence analysis, NK cells in the PARPi treatment group seemed to have a stronger antitumor effect, as manifested by a higher number of TINK and effector NK cells (marked by granzyme B [GZMB]) (Figure 1F and Supplemental Figure 2, B and C). To exclude the role of the synthetic lethal mechanism of PARPi in PPSM models (*BRCA1* deficient), we further used RM-1, a mouse PCa model with WT *BRCA1* (Supplemental Figure 2D). Similarly, PARPi still exhibited good tumor control of *BRCA1* WT PCa in immunocompetent mice (Figure 1G). In *Rag1*^–*/*–^ *γc*^–*/*–^ mice without T, B, and NK cells, however, PARPi lost its inhibitory effects on RM-1 and only produced a synthetic lethal antitumor effect in RM-1 *BRCA1* KO tumors (Figure 1H), suggesting that the immune system plays a critical role in PARPi treatment of tumors with nonhomologous recombination deficiency.

After treatment with PARPi, the proportion and number of TINK and GZMB^+^ NK were noticeably increased (Figure 1, I and J, and Supplemental Figure 2, E and G). Specific depletion of NK cells markedly attenuated the therapeutic efficacy of PARPi in RM-1 models, indicating that TINK cells are crucial for the antitumor effects of PARPi (Supplemental Figure 2H). Although the proliferation of TINK cells showed no significant change (Supplemental Figure 2, F and G), a reduction in peripheral blood NK cells (PBNKs) was observed in the mice, along with elevated levels of TNF-α and IFN-γ (Figure 1, K and L), suggesting that peripheral NK cells were activated during PARPi treatment and effectively chemotaxed into PCa.

### PARPi stimulate PCa cells to release CypA.

To determine whether PARPi reactivates NK cells in PCa through direct NK cell targeting or tumor–NK cell crosstalk, we conducted systematic investigations. RNA-seq revealed no significant alterations in NK cell expression of selected chemokine receptors and effector mediators under PARPi stimulation (Supplemental Figure 3, A and B), suggesting that PARPi does not directly act on NK cells. Intriguingly, NK cells cultured with conditioned media from PARPi-treated RM-1 cells exhibited enhanced activation (Supplemental Figure 3, C and D), suggesting that PARPi triggers PCa cells to secrete cytokine-like factors that activate NK cells.

**Figure 2:**
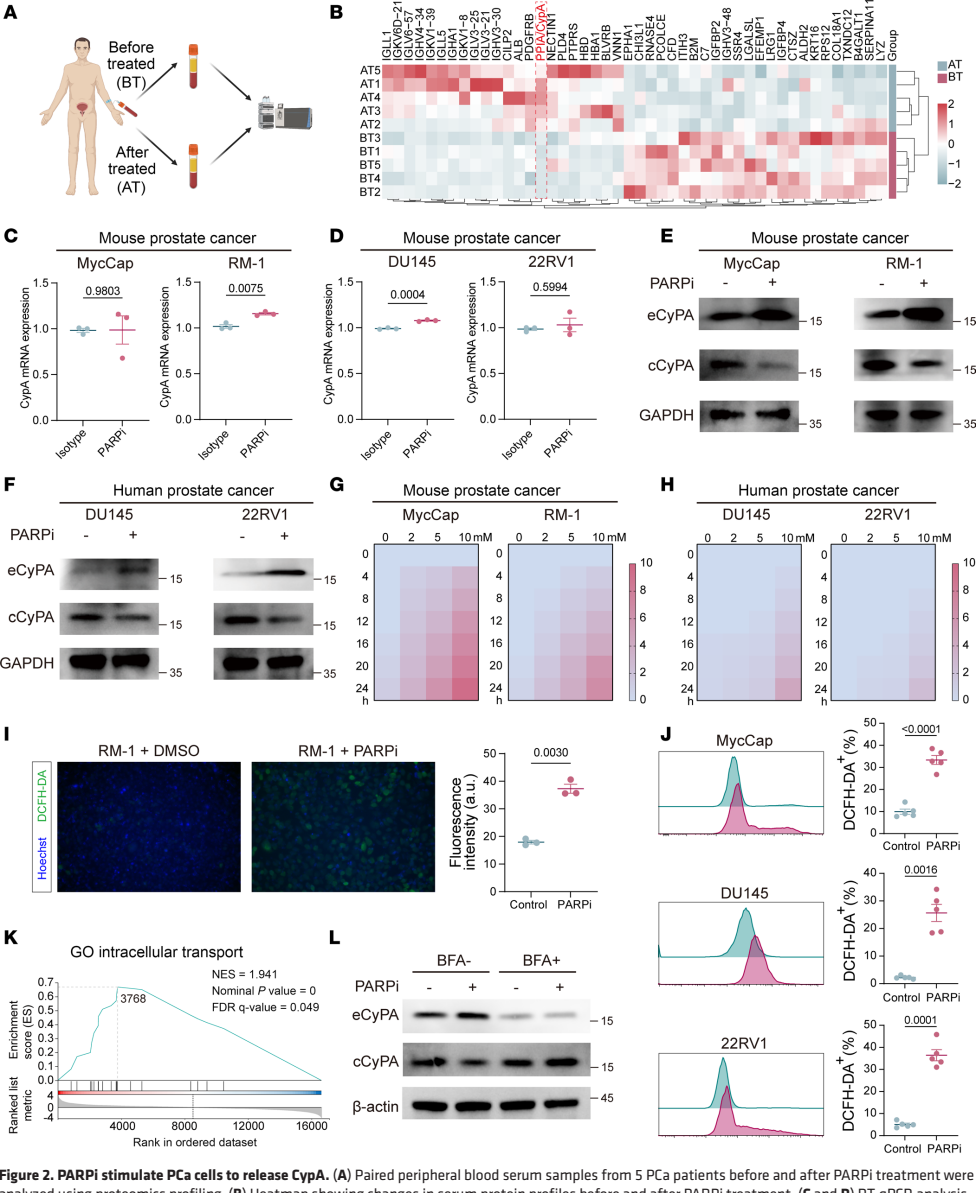
PARPi stimulate PCa cells to release CypA. (**A**) Paired peripheral blood serum samples from 5 PCa patients before and after PARPi treatment were analyzed using proteomics profiling. (**B**) Heatmap showing changes in serum protein profiles before and after PARPi treatment. (**C** and **D**) RT-qPCR analysis of PARPi-induced changes in CypA mRNA levels in murine PCa cell lines (**C**) and human PCa cell lines (**D**); *N* = 3 per group. (**E** and **F**) Western blot analysis of PARPi-induced changes in intracellular CypA (cCypA) and eCypA protein levels in murine (**E**) and human (**F**) PCa cell lines. (**G** and **H**) ELISA of PARPi-induced changes in CypA supernatant levels in murine (**G**) and human (**H**) PCa cell lines. (**I** and **J**) DCFH-DA (green) staining to detect PARPi-induced ROS levels in PCa cell lines RM-1 (**I**; *N* = 3) and MycCap/DU145/22RV1 (**J**; *N* = 5). Original magnification, ×100. (**K**) Gene Ontology enrichment analysis showing enhanced intracellular transport pathways in PCa cells after PARPi stimulation. (**L**) Western blot analysis of cCypA and eCypA levels after protein transport inhibition by Brefeldin A (BFA). Data are presented as mean ± SEM and were analyzed by Welch’s *t* test.

Thus, we performed proteomics analysis of peripheral blood serum from 5 CRPC patients before and after PARPi treatment (Figure 2A and Supplemental Figure 4A). Our results revealed significant activation of pathways related to immune response, lymphocyte-mediated immunity, and extracellular exosomes following PARPi therapy (Supplemental Figure 4, B and C). We next investigated the effects of PARPi therapy on human (DU145 and 22RV1) and mice (MycCaP and RM-1) PCa cell lines with different HRR gene statuses (Supplemental Figure 4D) (26). In vitro experiments using low-dose PARPi (nonapoptotic concentration) (Supplemental Figure 4E) reconfirmed the activation of DNA repair pathways, mismatch repair, homologous recombination, and DNA replication in PCa cells, as previously reported (Supplemental Figure 4F) (3). Of note, PARPi supplementation in the coculture medium of *BRCA*^WT^ RM-1 cells and NK cells markedly enhanced the tumor-killing ability of NK cells and maximized PARPi efficacies (Supplemental Figure 4, G and H), implying PARPi therapy could be used to treat PCa regardless of *BRCA* mutation status via a secretory crosstalk between NK cells and PCa cells. From the proteomics analysis, we identified cyclophilin A (CypA; a peptidyl-prolyl isomerase encoded by *Ppia*) as a prominently elevated factor and potential messenger mediating the crosstalk (Figure 2B). CypA is secreted extracellularly under oxidative stress/inflammation and serves as an inflammatory biomarker (27–29). Retrospective analysis of 5-year clinical specimens revealed lower serum CypA levels in patients with PCa versus those considered healthy controls, with inverse correlation to Gleason scores (Supplemental Figure 5, A and B). PARPi-induced CypA elevation was confirmed in murine models (Supplemental Figure 5C). Subcytotoxic PARPi treatment (1 μM, 48 h) induced intracellular CypA, which decreased with concurrent extracellular CypA (eCypA) accumulation across all lines (Figure 2, E and F), independent of CypA transcriptional changes (Figure 2, C and D) or *BRCA* status. Dose- and time-dependent CypA secretion was demonstrated (Figure 2, G and H). PARPi triggered ROS elevation in all cell lines (Figure 2, I and J), consistent with CypA’s role in oxidative stress response (30), alongside intracellular transport activation (Figure 2K). Brefeldin A (protein transport inhibitor) (31) substantially suppressed CypA release (Figure 2L), confirming vesicle-mediated secretion (32).

### CypA overexpression in tumors boosts NK cell chemotaxis and cytotoxicity.

**Figure 3:**
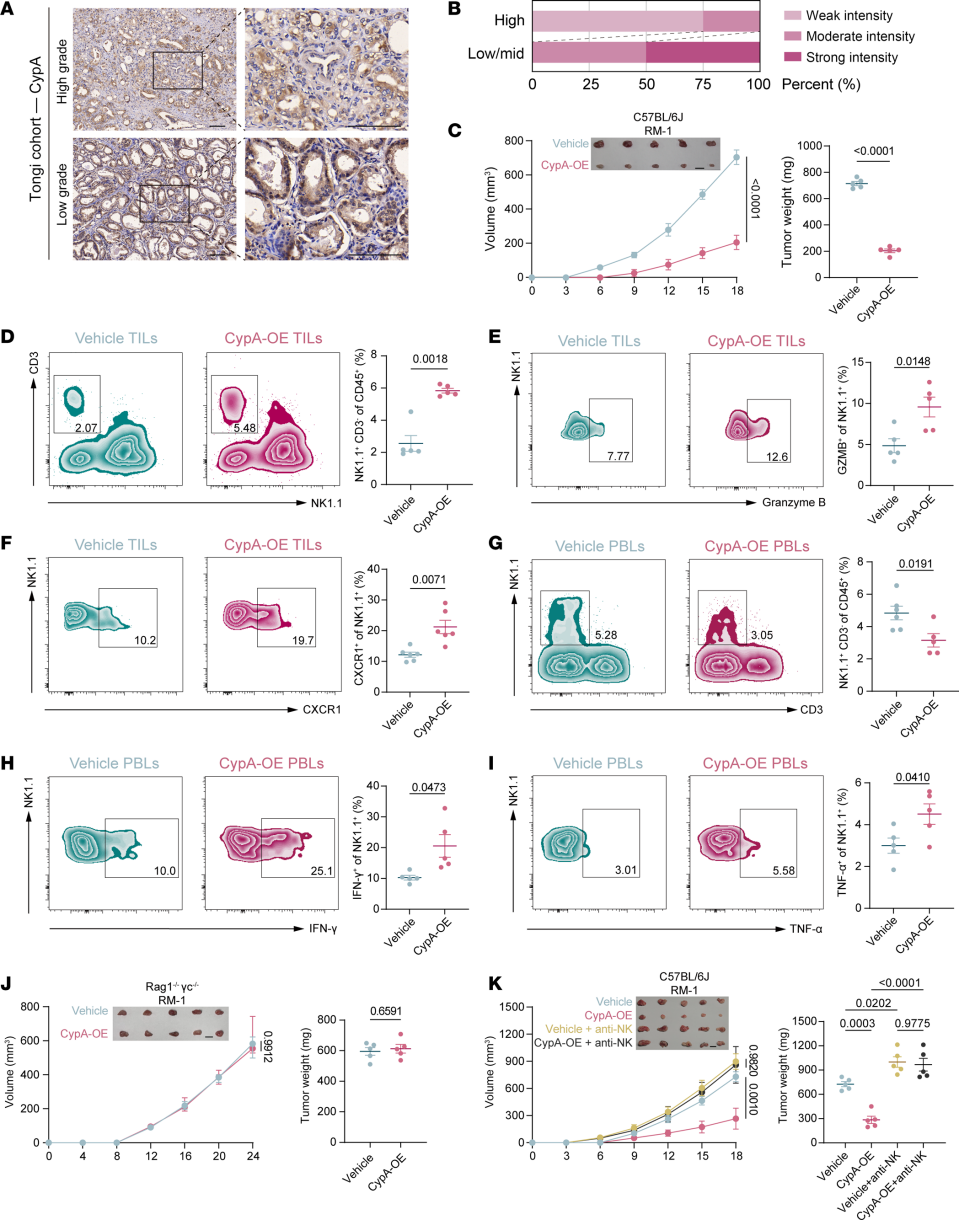
CypA overexpression in tumors boosts NK cell chemotaxis and cytotoxicity. (**A**) Immunohistochemical staining of CypA in tumor tissues from patients with high- and low-grade PCa, collected at Tongji Hospital. Scale bars: 100 μm. (**B**) Proportional statistics of NCR1 expression in patients with high-grade versus intermediate/low-grade PCa; *N* = 5 per group. (**C**) Tumor growth curves and weights in C57BL/6 mice inoculated with RM-1 (vehicle/CypA-OE) cell lines; *N* = 5 per group. Scale bars: 1 cm. (**D**) Flow cytometry analysis of NK cell proportions in tumors of mice bearing CypA-OE RM-1 cells; *N* = 5 per group. (**E** and **F**) Flow cytometry analysis of GZMB^+^ (**E**; *N* = 5) and CXCR1^+^ (**F**; *N* = 6) proportions in TINKs from mice bearing CypA-OE RM-1 cells. (**G**) Flow cytometry analysis of NK cell proportions in peripheral blood of mice bearing CypA-OE RM-1 cells; *N* = 5 per group. (**H** and **I**) Flow cytometry analysis of IFN-γ^+^ (**H**) and TNF^+^ (**I**) proportions in PBNKs from mice bearing CypA-OE RM-1 cells; *N* = 5 per group. (**J**) Tumor growth curves and weights in immunodeficient mice bearing CypA-OE tumors; *N* = 5 per group. Scale bars: 1 cm. (**K**) Tumor growth curves and weights in NK cell–depleted C57BL/6 mice inoculated with CypA-OE RM-1 cell lines; *N* = 5 per group; Scale bars: 1 cm. Tumor growth curve data are presented as mean ± SD and were analyzed by 2-way ANOVA with Tukey’s multiple-comparison test. Other data are presented as mean ± SEM and were analyzed by 1-way ANOVA (**K**) and Welch’s *t* test (**C**–**J**).

To further investigate the role of CypA in the immune microenvironment of PCa, we first constructed CypA overexpression (OE) cell lines, which exhibited increased protein expression levels both intracellularly and extracellularly (Supplemental Figure 5D). Analysis of PCa tissue specimens collected from Tongji Hospital revealed relatively lower CypA expression in high-grade PCa tissues (Figure 3, A and B). This differential expression pattern was similarly observed in the The Human Protein Atlas (https://www.proteinatlas.org/). (Supplemental Figure 5, E and F). In the PCa TCGA database, patient stratification based on CypA gene expression levels demonstrated that high CypA expression was associated with elevated activated NK cells through CIBERSORT analysis (Supplemental Figure 5, G and H).

Using 3 murine PCa cell lines (RM-1, MycCap, and PPSM) to model low-androgen receptor PCa (33), hormone-sensitive prostate cancer (34), and CRPC, respectively (25), we found that CypA-OE groups exhibited considerably slower tumor growth rates and reduced tumor weights compared with controls (Figure 3C and Supplemental Figure 6, A and B). Correspondingly, serum CypA levels were markedly elevated in these mice (Supplemental Figure 6C). Flow cytometry analysis of tumor tissues and peripheral blood (Supplemental Figure 6D) revealed increased proportions of TINKs and GZMB^+^ NK cells in CypA-OE tumors (Figure 3, D and E). Similar to PARPi-treated cohorts, CypA-OE groups showed decreased peripheral NK cell proportions but enhanced cytotoxic functionality, as evidenced by elevated IFN-γ^+^ NK and TNF-α^+^ NK cell frequencies (Figure 3, G–I). In human peripheral blood, CD56^dim^ NK cells constitute the predominant subset (~90%) and primarily mediate cytotoxic functions (35). These cells express the C-X-C motif chemokine receptor 1 (CXCR1), which has been reported to enhance CAR-NK cell infiltration into solid tumors (36). We observed upregulated CXCR1 expression in TINKs from CypA-OE groups (Figure 3F). Other major tumor-infiltrating lymphocyte (TIL) subpopulations (DCs, macrophages, CD4^+^ T cells, CD8^+^ T cells, and Tregs) and functional markers (PD-1 on CD8^+^ T cells and CD107a on CD8^+^ T cells) remained unaltered (Supplemental Figure 6, E–J). To validate the critical roles of NK cells in CypA-mediated tumor suppression, *Rag1*^–*/*–^ *γc*^–*/*–^ mice were used and showed abolished CypA-mediated tumor inhibition (Figure 3J). In immunocompetent C57BL/6J mice, selective depletion experiments demonstrated preserved tumor-suppressive effects after CD8^+^ T cell depletion (Supplemental Figure 7, A and B), whereas NK cell depletion completely abrogated CypA-mediated tumor suppression (Figure 3K and Supplemental Figure 7, C and D). These findings collectively indicate that CypA exerts its antitumor effects through functional NK cell–dependent mechanisms.

### CypA enhances mitochondrial oxidative phosphorylation in NK cells.

**Figure 4:**
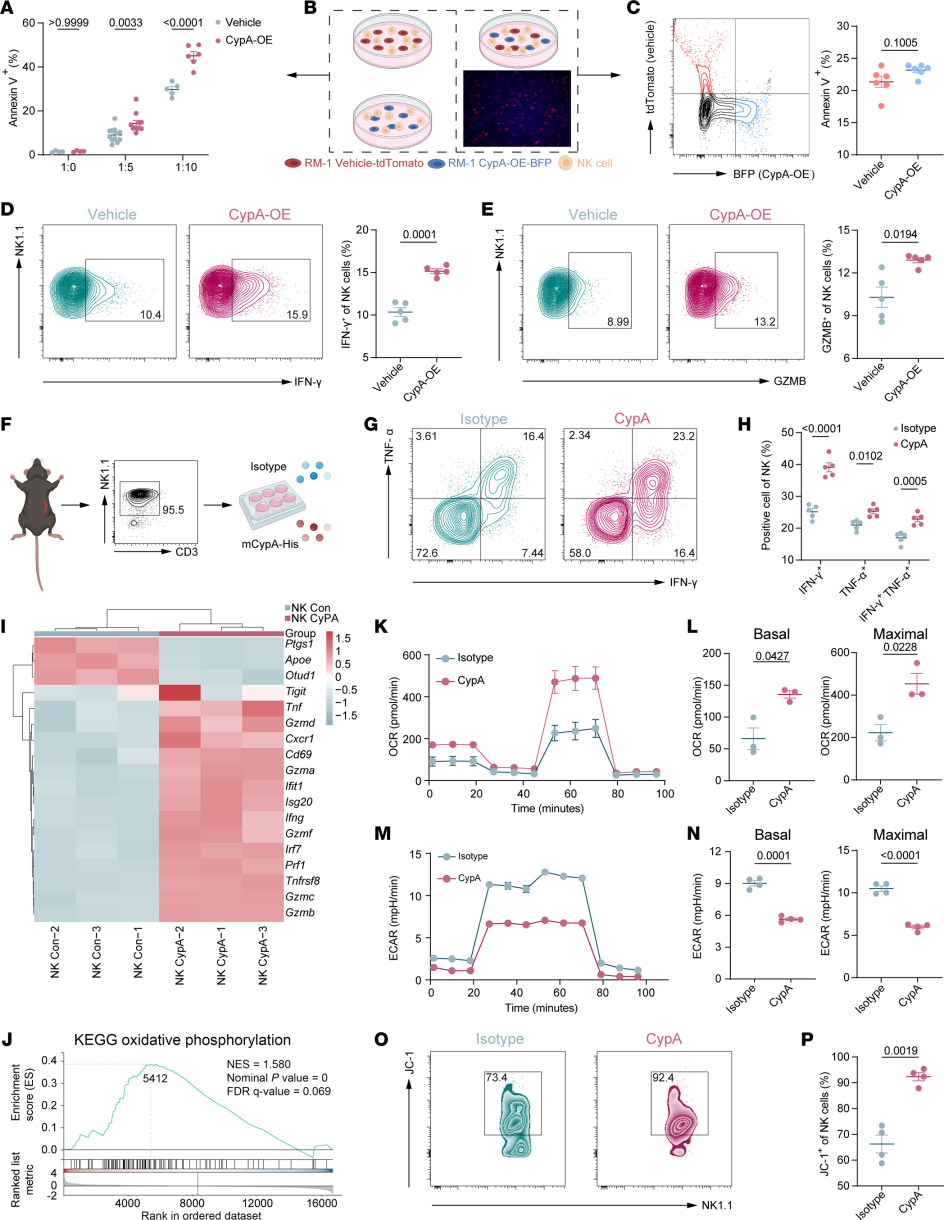
CypA enhances mitochondrial oxidative phosphorylation in NK cells. (**A**) Apoptosis levels of RM-1 cells (transfected with CypA plasmid or vehicle control) cocultured with mouse NK cells at ratios of 1:0, 1:5, and 1:10 for 24 hours, as assessed by annexin V/PI staining. (**B**) Experimental design of the tumor cell–NK cell coculture killing assay. Original magnification, ×100. (**C**) Coculture system of tdTomato-labeled (red) RM-1–vehicle control cells and BFP-labeled (blue) RM-1–CypA-OE cells at equal numbers. Apoptosis rates of fluorescently labeled tumor cells after 24 h NK cell–mediated killing, as determined by flow cytometry; *N* = 6 per group. BFP, blue fluorescent protein. (**D** and **E**) IFN-γ (**D**) and GZMB (**E**) levels in NK cells cocultured with RM-1 cells (as described in **A**); *N* = 5. (**F**) Purified mouse splenic NK cells (purity validated) cultured with recombinant CypA protein or isotype control. (**G** and **H**) Flow cytometry analysis of TNF-α and IFN-γ levels (**G**) and statistical quantification (**H**) in NK cells treated with recombinant CypA or isotype control; *N* = 5. (**I**) RNA-seq analysis of transcriptomic changes in NK cells exposed to CypA. (**J**) KEGG enrichment analysis showing upregulated oxidative phosphorylation in NK cells. (**K**–**N**) Cellular energy metabolism analysis: oxygen consumption rate (**K** and **L**) and extracellular acidification rate (**M** and **N**) in NK cells. (**O** and **P**) Mitochondrial membrane potential assay evaluating aerobic respiration capacity (**O**) and statistical results (**P**) in NK cells; *N* = 4. Data are presented as mean ± SEM and were analyzed by Welch’s *t* test.

We isolated mouse NK cells and performed coculture experiments with RM-1 vehicle or RM-1 CypA-OE cells. Flow cytometry showed that RM-1 CypA-OE cells underwent increased apoptosis during NK cell attack (Figure 4, A and B, and Supplemental Figure 8A). To determine whether elevated eCypA accounted for this difference, we fluorescently labeled the cells (tdTomato for vehicle and blue fluorescent protein for CypA-OE), mixed them equally, and cocultured them with NK cells. In this shared environment, the disparity in apoptosis was eliminated (Figure 4, B and C), indicating that the differential apoptosis observed in separate cocultures did not result from alterations in direct immune synapse formation between NK cells and their respective target cells (37). This implicates a secreted factor within the common culture environment in regulating NK cell cytotoxicity. Further analysis revealed that NK cells cocultured with CypA-OE cancer cells exhibited upregulated expression of antitumor effector molecules (Figure 4, D and E). However, RM-1–OVA–CypA-OE cells failed to promote any further activation of OT-1 mouse-derived CD8^+^ T cells or enhance their mediated antitumor effects (Supplemental Figure 8, B–E).

To validate the role of CypA in vivo, an anti-CypA mAb (28) and cyclosporin A (CsA; an immunosuppressant that binds and inhibits CypA) (38) were intraperitoneally injected into tumor-bearing mice. Both the antibody and the inhibitor abolished the tumor-suppressive effect of CypA-OE (Supplemental Figure 9, A and B). Additionally, the immunosuppressant CsA also suppressed the infiltration and effector function of TINK cells (Supplemental Figure 9C) (39, 40). Direct NK cell activation by CypA was confirmed using isolated murine NK cells cultured with recombinant CypA protein (Figure 4F). CypA stimulation elevated TNF-α and IFN-γ production (Figure 4, G and H) and upregulated transcripts associated with effector function and chemokine receptors, while the surface receptors NKG2A, NKG2D, and NKp46 remained unchanged (Figure 4I and Supplemental Figure 9D). GSEA and KEGG analysis revealed significant enrichment of oxidative phosphorylation pathways (Figure 4J and Supplemental Figure 9E). Functional assays demonstrated that CypA enhanced mitochondrial aerobic respiration and reduced extracellular acidification (Figure 4, K–N). Measurement with the JC-1 dye demonstrated that CypA increases the mitochondrial membrane potential in NK cells (Figure 4, O and P), suggesting higher mitochondrial activity. Taken together, these results confirmed that CypA enhances mitochondrial oxidative phosphorylation in NK cells.

### ANXA6 is identified as a CypA receptor mediating FPR1 signaling in NK cells.

**Figure 5:**
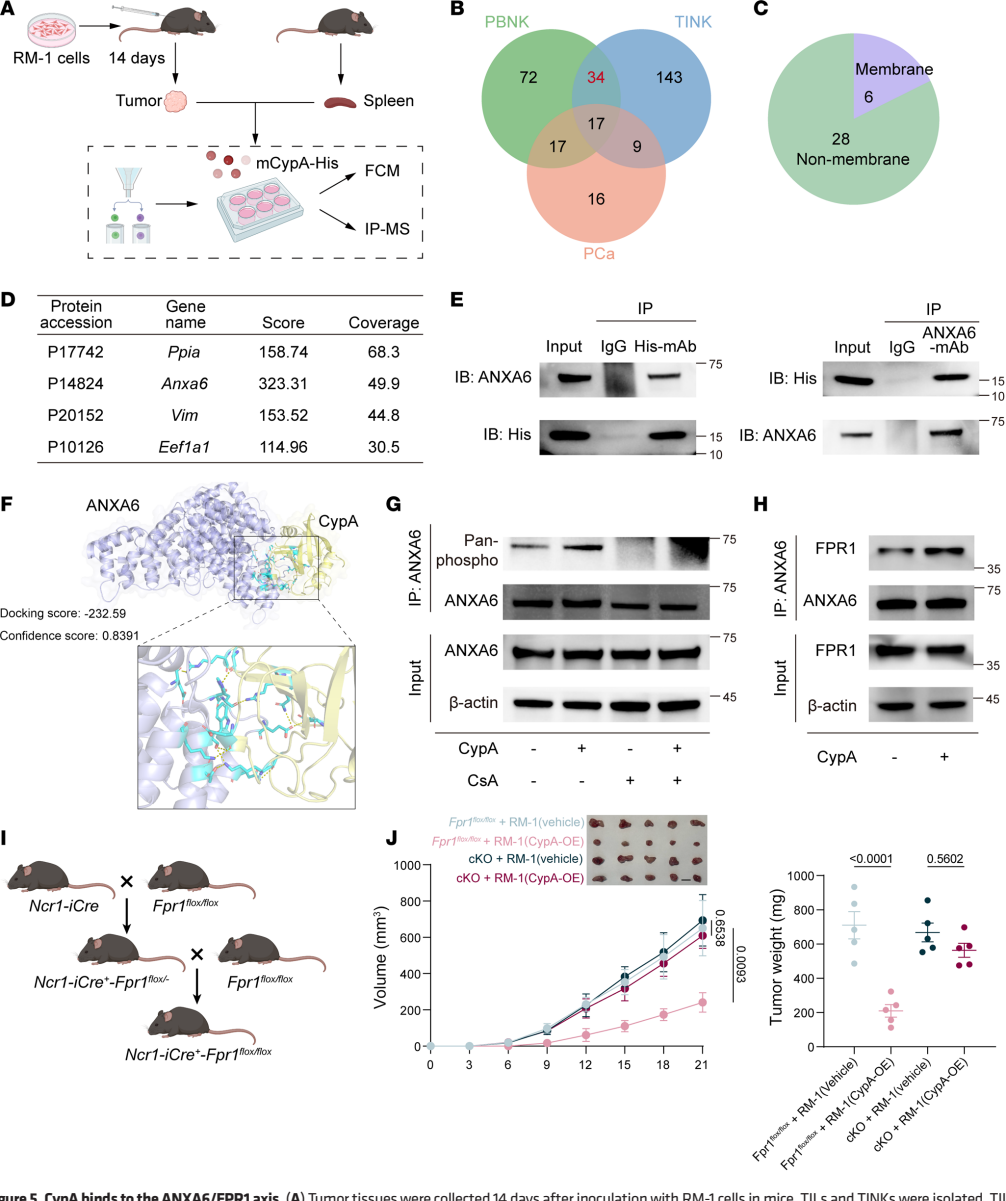
CypA binds to the ANXA6/FPR1 axis. (**A**) Tumor tissues were collected 14 days after inoculation with RM-1 cells in mice. TILs and TINKs were isolated. TILs were cultured with recombinant proteins in vitro, and recombinant protein tags were detected via flow cytometry. TINKs were cultured with recombinant proteins in vitro, and binding proteins were identified by IP-MS. FC, flow cytometry. (**B**) Venn diagram showing proteins with IP-MS scores ≥ 100 in TINKs, PBNKs, and PCa cells. (**C**) Proportion of membrane proteins versus nonmembrane proteins among overlapping proteins from **B**. (**D**) Membrane protein profiles identified by IP-MS. (**E**) Co-IP demonstrating mutual binding between ANXA6 and CypA-His. IB, immunoblotting. (**F**) Protein docking prediction between ANXA6 and CypA proteins using AlphaFold3. (**G**) Western blot analysis of ANXA6 expression and phosphorylation levels in NK cells treated with CypA and/or CsA. (**H**) Co-IP analysis of ANXA6 and FPR1 interaction in CypA-treated NK cells. (**I**) *Fpr1^fl/fl^* mice were crossed with the *Ncr1-iCre* transgenic mice to generate the NK cell–specific *Fpr1*-KO mice, which are denoted as *Ncr1-iCre^+^-Fpr1^fl/fl^*. (**J**) Tumor growth curves and tumor weights in *Fpr1^fl/fl^* and *Ncr1-iCre^+^-Fpr1^fl/fl^* (cKO) mice inoculated with RM-1 vehicle or CypA-OE cells, respectively; *N* = 5 per group. Tumor growth curve data are presented as mean ± SD and were analyzed by 2-way ANOVA with Tukey’s multiple-comparison test. Tumor weight data are presented as mean ± SEM and were analyzed by 1-way ANOVA.

To investigate the mechanism of CypA’s interaction with NK cells, we incubated TILs and spleen lymphocytes with tagged recombinant CypA protein (Figure 5A), which showed preferential binding to NK cells (Supplemental Figure 10, A and B). Subsequent immunoprecipitation–mass spectrometry (IP-MS) analysis of TINKs, PBNKs, and PCa cells cocultured with recombinant CypA identified membrane-associated proteins with interaction scores ≥ 100 (Figure 5, B and C). Annexin A6 (ANXA6), a protein critical for membrane repair and signal transduction, emerged as a top candidate (Figure 5D) (41). Coimmunoprecipitation (co-IP) assays using CypA-His–treated NK cells confirmed direct binding between CypA and ANXA6 (Figure 5E). In addition, it corroborated the CypA-ANXA6 docking prediction (Figure 5F).

Further validation revealed higher ANXA6 expression in both human and murine NK cells compared with T cells (Supplemental Figure 10, C and D), while PCa cells showed lower ANXA6 expression compared with other tumor cells, including bladder cancer, colorectal, renal, and hepatocellular carcinoma cell lines (Supplemental Figure 10, E and F). Given CypA’s reported phosphorylation activity and ANXA6’s phosphorylation motifs (42), we observed increased global phosphorylation in NK cells (Supplemental Figure 10, G and H) and specifically elevated ANXA6 phosphorylation following CypA stimulation (Figure 5G). While the homologous family member ANXA1 activates FPR1-dependent downstream pathways (43), the role of ANXA6 remained unclear. Strikingly, phosphorylated ANXA6 demonstrated enhanced binding to FPR1 (Figure 5H). To further verify whether CypA activates NK cells through the downstream pathway of FPR1 within NK cells, we constructed a *Ncr1-iCre-Fpr1-loxp* NK cell conditional KO mouse model (Figure 5I and Supplemental Figure 10I). Experiments with tumor-bearing mice demonstrated that the tumor-suppressive effect mediated by CypA depends on FPR1 expression on NK cells (Figure 5J and Supplemental Figure 10J).

### FPR1 mediates AKT and ERK1/2 phosphorylation to enhance NK cell efficacy.

**Figure 6:**
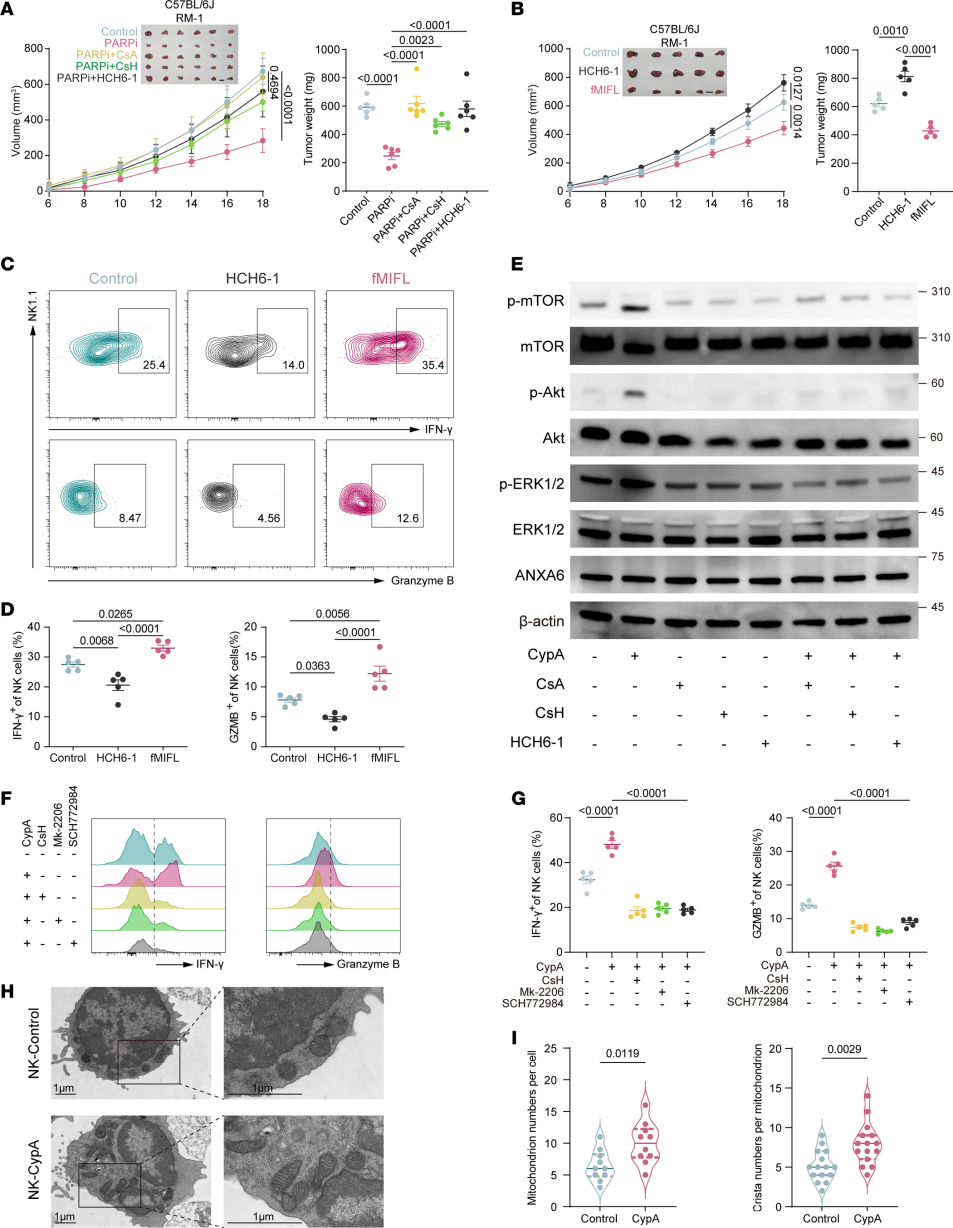
FPR1 mediates AKT and ERK1/2 phosphorylation to enhance NK cell efficacy. (**A**) Tumor growth curves and weights in PARPi-treated tumor-bearing mice coadministered with CypA inhibitor CsA and FPR1 inhibitors CsH and HCH6-1; *N* = 6 per group; scale bars: 1 cm (**B**) Tumor growth curves and weights in C57BL/6 mice bearing RM-1 tumors treated with FPR1 agonist fMIFL or inhibitor HCH6-1; *N* = 5 per group; scale bars: 1 cm (**C** and **D**) IFN-γ and GZMB expression levels in TINKs (**C**) and their quantification (**D**) under HCH6-1 or fMIFL treatment; *N* = 5 per group. (**E**) Western blot analysis of ERK- and AKT-related pathway activation in NK cells treated with CypA, CsA, CsH, or HCH6-1 in vitro. (**F** and **G**) Flow cytometry analysis of IFN-γ and GZMB expression (**F**) and quantification (**G**) in NK cells treated with FPR1 inhibitor CsH, AKT inhibitor MK-2206, or ERK inhibitor SCH772984; *N* = 5 per group. (**H**) Transmission electron microscopy images showing mitochondrial structure and crista alterations in NK cells stimulated with CypA. Scale bars: 1 μm. (**I**) Statistical analysis of mitochondrial number (*N* = 10) and crista count (*N* = 15) in NK cells. Tumor growth curve data are presented as mean ± SD and were analyzed by 2-way ANOVA with Tukey’s multiple-comparison test. Other data are presented as mean ± SEM and were analyzed by 1-way ANOVA (**A**, **B**, **D**, and **G**) and Welch’s *t* test (**I**).

To investigate whether FPR1 and its downstream signaling pathways are critical for PARPi-mediated activation of NK cell effector functions, we employed 2 FPR1 inhibitors: CsH (a CsA analog with higher inhibitory potency) (44) and HCH6-1 (45). Both inhibitors abolished the therapeutic efficacy of PARPi in RM-1 tumors (Figure 6A). Consistent with previous reports, treatment with the FPR1 agonist fMIFL considerably reduced tumor growth (Figure 6B) and enhanced the antitumor activity and chemotactic migration of TINKs (Figure 6, C and D) (46, 47).

Further mechanistic studies revealed that CypA robustly activates the ERK and AKT/mTOR pathways downstream of FPR1 (Figure 6E) (46, 48, 49). Pharmacological inhibition of FPR1 (CsH), AKT (MK-2206) (50), or ERK (SCH772984) (50, 51) abrogated CypA-induced activation of NK cell antitumor responses (Figure 6, F and G). Notably, AKT/mTOR signaling critically regulates mitochondrial metabolic reprogramming, a finding corroborated by the increased mitochondrial content and crista density observed in CypA-stimulated NK cells (Figure 6, H and I).

### The combination of PARPi with adoptive NK therapy reduces CRPC progression.

NK cells exhibit potent tumoricidal activity and robust infiltration capacity during early tumorigenesis. However, as solid tumors expand, the developing stromal barrier progressively hinders NK cell infiltration. Even infiltrated NK cells rapidly lose effector functions and fail to sustain antitumor responses (19, 52). Longitudinal analysis of tumors harvested on days 7, 9, 11, 13, and 15 after implantation revealed peak NK cell infiltration density on day 7 (when tumors just formed small nodules), with a sharp decline in TINK abundance and concomitant reduction in GZMB^+^ NK cells over time (Supplemental Figure 11, A and B).

Based on these findings, we adoptively transferred TINKs isolated from untreated or PARPi-treated tumor-bearing mice into recipient mice with established tumors (Supplemental Figure 11C). While naive TINKs showed no therapeutic impact, PARPi-primed TINKs substantially suppressed tumor growth (Supplemental Figure 11D), demonstrating enhanced intratumoral infiltration and cytotoxicity (Supplemental Figure 11, E and F).

**Figure 7:**
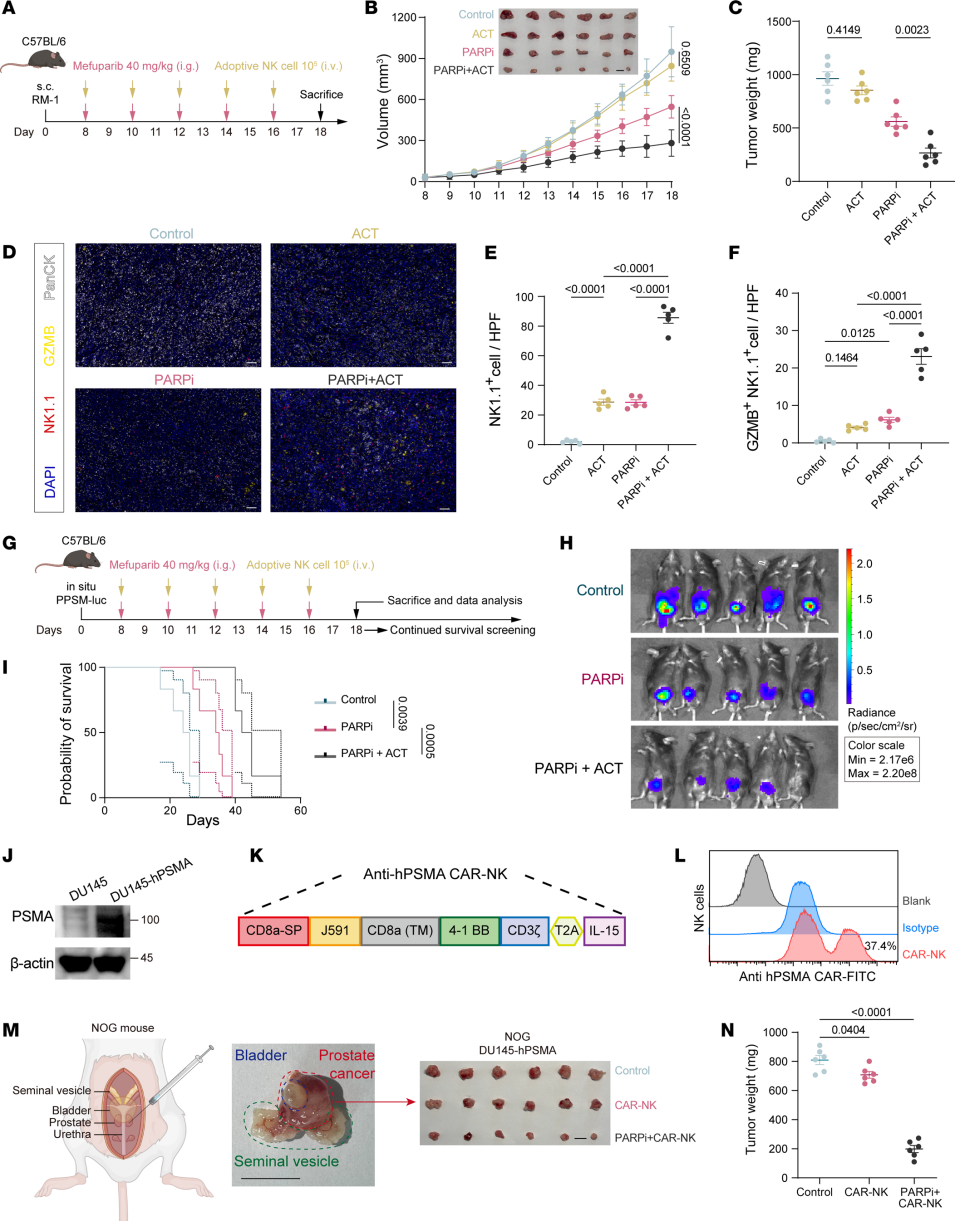
The combination of PARPi with adoptive NK therapy inhibits CRPC growth and prolongs survival. (**A**) Mice bearing RM-1 tumors were treated with mefuparib and adoptive transfer of 10^5^ NK cells every 2 days from day 8. Mice were euthanized on day 18 for tumor collection. (**B** and **C**) Tumor growth curves (**B**) and weights (**C**) in mice treated with adoptive NK cell therapy, PARPi, or combination therapy; *N* = 6; scale bars: 1 cm. (**D**) Immunofluorescence of tumors from mice treated with adoptive NK cells, PARPi, or combination therapy, showing DAPI (blue), NK1.1 (red), GZMB (yellow), and PanCK (white). Scale bars: 50 μm. (**E** and **F**) Quantification of average NK1.1^+^ cells (**E**) and GZMB^+^ NK1.1^+^ cells (**F**) per high-power field. Data are based on average counts from 5 random fields per sample; *N* = 5. (**G**) Mice bearing orthotopic PPSM tumors received mefuparib and 10^5^ NK cells every 2 days from day 8. Mice were euthanized on day 18 for analysis or retained for survival observation. (**H**) In vivo small animal imaging of orthotopic prostate tumor growth on day 18. (**I**) Survival curves of orthotopic PCa mouse models. (**J**) Western blot confirming hPSMA expression in DU145 cells. (**K**) Design of CAR-NK cells targeting human PSMA. SP, signal peptide; TM, transmembrane. (**L**) Representative flow cytometry analysis showing the transduction efficiency of CAR-NK cells. (**M**) Establishment of the DU145-hPSMA in situ PCa implantation model. Subsequently, tumor-bearing mice were treated with mefuparib and 10^5^ CAR-NK cells every 2 days from day 8. Mice were euthanized on day 24 for tumor collection. (**N**) Tumor weight in DU145-hPSMA–bearing mice treated with PARPi and/or CAR-NK cells; *N* = 6. Tumor growth curve data are presented as mean ± SD and were analyzed by 2-way ANOVA with Tukey’s multiple-comparison test. Survival curves were analyzed by a log-rank (Mantel-Cox) test. Other data are presented as mean ± SEM and were analyzed by 1-way ANOVA.

We subsequently developed a combination therapy integrating PARPi with adoptive NK cell transfer (Figure 7A). Monotherapy with adoptive NK cells alone failed to inhibit tumor progression, whereas the combinatorial approach elicited potent antitumor effects (Figure 7, B and C, and Supplemental Figure 12A), accompanied by increased intratumoral GZMB^+^ NK cells (Figure 7, D–F). Using PPSM-luciferase cells to model in situ CRPC (Figure 7G), we observed delayed tumor progression and prolonged survival in mice receiving PARPi plus adoptive cell therapy (Figure 7, H and I). Notably, adoptive NK cell transfer synergized with PARPi in both amplifying NK cell cytotoxicity and expanding the TINK population (Supplemental Figure 12, B–G).

To further evaluate the therapeutic potential of this combination strategy for patients with PCa, we stably introduced the human prostate-specific membrane antigen (hPSMA) sequence into the *BRCA*^WT^ human metastatic PCa cell line DU145 (Figure 7J). PSMA is highly expressed in PCa cells of 95% of patients and has been employed as a clinical targeting marker (53, 54). Using the reported J591 antibody to target hPSMA (55), we constructed corresponding CAR-NK cells based on an established system (Figure 7, K and L) (56). Subsequently, DU145-hPSMA cells were used to establish an orthotopic PCa model in NOG mice. The results showed that treatment with PARPi combined with CAR-NK cells effectively suppressed prostate tumor growth (Figure 7, M and N). These data suggest that the combination of PARPi and CAR-NK therapy holds potential value for the treatment of advanced PCa.

## Discussion

PCa is considered a cold tumor in response to anti–PD-1 therapy due to its heterogenous characteristics, loss of MHC-I antigens, and an immunosuppressive microenvironment enriched with Tregs, myeloid-derived suppressor cells, and tumor-associated macrophages (57). This environment hinders the inflammatory infiltration of cytotoxic T lymphocytes and NK cells. Additionally, prostaglandins (58, 59) and other substances secreted by PCa further impair the antitumor function of intratumoral cytotoxic immune cells.

Although NK cells hold potential as a therapeutic target for PCa immunotherapy, a deeper understanding of the mechanisms underlying NK cell dysfunction in cancer is required to overcome immunosuppressive factors. A critical factor contributing to this functional suppression is the metabolic dysregulation observed in NK cells. An increasing number of therapeutic interventions are targeting key metabolic pathways in NK cells, including mTORC1 signaling (60), oxidative phosphorylation (61, 62), and glycolysis (63), to counteract the immunosuppressive hypoxic and highly acidic TME (64, 65).

Tumor-secreted proteins dynamically modulate immune cell states and functions through multiple mechanisms: recruiting immune cells via chemokine secretion (66), suppressing antitumor immunity through soluble immune checkpoints (sPD-L1, sCTLA-4, and sCD109, reviewed in refs. 67–69), and enhancing CD8^+^ T/NK cell activity via secreted PTEN-mediated polarization of tumor-associated macrophages toward inflammatory phenotypes (70). Drug-induced alterations in tumor-secreted proteins suggest that exploiting therapy-triggered changes in tumor secreted proteins could create like cures like therapeutic opportunities (71, 72).

Our findings show that PARPi treatment can effectively revitalize TINKs, promoting their recruitment into PCa and restoring their antitumor function. In tumor cells lacking *BRCA* mutations or *BRCA*-like phenotypes, PARPi mediated ROS suppression has been proposed as a potential therapeutic target for PARPi (73). Mechanistically, PARPi-induced elevation of ROS levels in PCa cells triggers the secretion of intracellular CypA into the TME. eCypA has been previously identified as a proinflammatory mediator that recruits and activates lymphocytes, participating in pathophysiological processes (74, 75). Here, we found that after PARPi treatment, eCypA levels substantially increased in the peripheral blood serum of both human patients and mice, stimulating the chemotaxis of NK cells into prostate tumors. Furthermore, eCypA binds to and promotes the phosphorylation of ANXA6 on NK cells, activating the downstream FPR1 signaling pathway and driving AKT/mTOR-mediated metabolic reprogramming in NK cells (76), thereby enhancing their antitumor efficacy.

Unlike T cells, NK cells are not restricted by MHC and directly kill target cells by binding to surface ligands (77). Adoptive NK cell therapy has demonstrated remarkable efficacy in hematologic malignancies (78), with a favorable safety profile and reduced risk of cytokine release syndrome or immune rejection. Therefore, optimizing NK cell therapy and extending its application to PCa treatment holds great promise. However, in previous studies, adoptive NK cells exhibited limited efficacy in solid tumors, likely due to dense tumor tissue, high fibrosis, and poor infiltration. Even when a small number of NK cells successfully infiltrate solid tumors, they quickly become dysfunctional due to the immunosuppressive microenvironment, failing to exert effective antitumor effects (52). Based on these findings, we explored the combination of PARPi and adoptive NK cell transfer therapy. Using subcutaneous xenograft and orthotopic CRPC mouse models, we observed that the combination therapy substantially increased the proportion and number of TINKs. Moreover, compared with the suppressed NK cells in the original PCa microenvironment, NK cells under the combination strategy exhibited restored antitumor activity. Finally, the combination of PARPi and CAR-NK therapy demonstrated promising efficacy against PCa, highlighting the potential of this therapeutic strategy.

This study has some limitations. The specific interaction between ANXA6 and FPR1 requires further investigation, which could guide the modification of NK cells or the design of clinically applicable CAR-NK therapies. Moreover, given the currently limited clinical indications for PARPi in PCa, paired pre- and posttreatment samples from patients receiving neoadjuvant PARPi are lacking. This gap hinders a comprehensive analysis of the TME landscape following PARPi therapy. Furthermore, well-designed clinical trials evaluating PARPi combined with CAR-NK in advanced PCa are warranted to fully assess the real-world efficacy and adverse event profile of this combinatorial approach.

In conclusion, our research addresses the challenges in current treatments for advanced PCa: the limited applicability of PARPi, poor response to immune checkpoint inhibitors, and the weak performance of NK cells in solid tumors. The combination of PARPi and adoptive NK cell transfer may expand the indications for PARPi and enhance immunotherapeutic efficacy in PCa treatment.

## Methods

### Sex as a biological variable.

Our study of PCa involved the use of male mice, as it is a male-exclusive disease. The relevance of these findings to female mice is unknown.

### Mice.

6- to 8-week-old male WT C57BL/6J mice, FVB mice, and NOG mice were purchased from Charles River Co., Ltd. *Ncr1-iCre* mice and *Fpr1^fl/fl^* mice were purchased from Cyagen Biosciences. OT-I mice were obtained from The Jackson Laboratory. *Rag1*^–*/*–^ *γc*^–*/*–^ mice were provided in-house. All animals were housed in a specific pathogen–free environment.

### Cell lines.

The murine PCa cell line RM-1 was obtained from ATCC. RM-1, RM-1-OVA (stably transfected with chicken ovalbumin), DU145, DU145-hPSMA, and 22RV1 were cultured in complete RPMI-1640 medium (Gibco) supplemented with 10% FBS. The murine PCa cell lines MycCap and PPSM were provided by Jun Qin’s laboratory (25). MycCap cells were maintained in basic DMEM (Gibco) supplemented with 10% FBS. PPSM and PPSM-Luc (stably transfected with firefly luciferase) were cultured in advanced DMEM supplemented with GlutaMAX (Gibco). The PPSM cell lines were cultured in accordance with the protocols established for prostatic organoids, as previously documented in the scientific literature (79). Murine NK cells were isolated from spleens and cultured in DMEM (10% FBS) supplemented with 10 ng/mL IL-2 and 100 ng/mL IL-15. All cells were cultured at 37°C with 5% CO_2_ atmosphere. Mycoplasma testing was routinely performed.

### Tumor models and treatments.

For subcutaneously inoculated tumors, 1 × 10^6^ RM-1, MycCap, or PPSM tumor cells were suspended in 100 μL PBS and injected subcutaneously into the right flank of mice. At 7 or 8 days after tumor injection, when the tumor volume reached about 50 mm^3^, the mice were randomly grouped to receive various treatments. The PARPi treatment used mefuparib (MedChemExpress), which was administered by gavage at a dose of 40 mg/kg per mouse (once every 2 days). Enzalutamide (MedChemExpress) was administered by gavage at a dose of 100 mg/kg per mouse (once a day). The drugs for gavage were dissolved as follows: 5% Tween 80, 10% DMSO, 40% PEG300, and 45% saline. Docetaxel (MedChemExpress) was injected via the tail vein at a dose of 10 mg/kg per mouse (once a week). Anti–PD-1 (BeiGene) was administered by intraperitoneal injection at a dose of 200 mg/kg per mouse (once every 3 days). The injected drugs were dissolved in saline. Subcutaneous tumors were measured for the long and short diameters using vernier calipers, and the volume was calculated according to the formula of half the product of the length and the square of the width. When the long diameter of the tumor reached 2 cm or the tumor volume reached 2,000 mm^3^, the experiment was terminated according to ethical requirements.

For orthotopic PCa, after opening the abdominal cavity of male mice, the prostate gland was located under the bladder, and 1 × 10^6^ PPSM-luc or DU145-hPSMA tumor cells were suspended in 50 μL PBS and injected into the prostate gland. At 18 to 20 days after tumor injection, 150 mg/kg of d-luciferin, potassium salt (Yeasen) was injected into the abdominal cavity of the mice, and in vivo imaging analysis was performed 10 minutes after injection into the body.

For in vivo depletion of immune cells, 200 μg per mouse of anti-mouse NK-1.1 (Bio X Cell) and anti-mouse CD8 (Bio X Cell) antibodies were administered intraperitoneally 2 days before tumor injection, followed by injections every 3 days.

For in vivo CypA blockade, anti-CypA mAb (BGI Genomics) was intraperitoneally administered at a dose of 200 μg per mouse on day 1 after tumor inoculation, followed by weekly injections thereafter (28).

For adoptive NK cell therapy, primary mouse NK cells were cultured in vitro with IL-2 (20 ng/mL) and IL-15 (50 ng/mL) for 3 days, then the 1 × 10^5^ NK or CAR-NK cells were suspended in 100 μL PBS and injected into the mice via the tail vein.

### RNA-seq and proteomics analysis.

Total RNA was extracted from cells using TRIzol, and sequencing was performed by BGI using the DNBSEQ platform. Clean data were aligned to the reference genome using Bowtie2 (v2.3.4.3). Gene expression quantification was conducted using RSEM (v1.3.1) software, and a clustering heatmap of gene expression levels across different samples was generated using pheatmap (v1.0.8). Differential gene expression analysis was performed using DESeq2 (v1.4.5), with the criteria set at *q* value ≤ 0.05 or FDR ≤ 0.001. For serum protein samples, proteins were extracted, digested with protease, and desalted. The resulting peptide mixtures were analyzed using liquid chromatography–tandem mass spectrometry. The obtained mass spectrometry data were searched against a protein database for identification and relative quantification of the proteins in the samples.

### Protein interaction assays and protein docking.

The CypA-His recombinant protein (200 ng/mL) was added to the culture medium of NK cells in vitro and incubated for 6 hours. The cells were then washed and centrifuged to remove unbound recombinant protein. Cellular proteins were extracted using the membrane and cytosol protein extraction kit (Epizyme), followed by overnight incubation with anti-His–conjugated beads. After washing the beads on a magnetic rack, they were ready for IP-MS analysis, or they could be denatured at room temperature (RT) with loading buffer for Western blot analysis. Protein sequences were obtained from the UniProt database (P17742 for mouse PPIA/CypA and P14824 for mouse ANXA6). AlphaFold3 (https://alphafoldserver.com/) was used for de novo modeling to generate complete 3D structural models of CypA and ANXA6 proteins. The interaction forms between the 2 proteins were simulated using PyMOL (https://pymol.org).

### Western blot analysis.

Total protein was extracted using RIPA lysis buffer (Servicebio) supplemented with protease and phosphatase inhibitors (Roche). Protein concentrations were quantified via BCA assay. Equal amounts of protein were separated by SDS-PAGE on 10% polyacrylamide gels and transferred onto PVDF membranes (Millipore). Membranes were blocked with 5% nonfat milk in TBST for 1 h at RT and then incubated overnight at 4°C with antibodies. After washing with TBST, membranes were incubated with HRP-conjugated secondary antibodies for 1 h at RT. Protein bands were visualized using ECL substrate and imaged with ChemiDoc MP Imaging System (Bio-Rad).

### Immunohistochemistry analysis.

Human tumor tissue samples were fixed in 10% neutral-buffered formalin for 24–48 h, embedded in paraffin, and sectioned at 4 μm thickness using a microtome. After deparaffinization in xylene and rehydration through a graded ethanol series, antigen retrieval was performed by heating in citrate buffer (pH 6.0). Subsequently, sections were blocked with 5% BSA for 1 h at RT and then incubated overnight at 4°C with primary antibody. Following washes with PBST (PBS containing 0.1% Tween 20), sections were incubated with HRP-conjugated polymer secondary antibody for 1 h at RT. Signals were developed using DAB chromogen for 5 min under microscopic control, followed by hematoxylin counterstaining, dehydration, and mounting.

### Flow cytometry analysis.

Tumor tissue blocks were digested with collagenase and placed in a grinder to prepare a single-cell suspension. Mouse lymphocytes were isolated using 38% Percoll (Biosharp), followed by erythrocyte lysis (Solarbio). A portion of the cells was resuspended in complete culture medium, and cell stimulation cocktail (BD Biosciences) was added. The cells were then incubated in a cell culture incubator for 6–8 hours. Initially, the cells were stained with surface marker antibodies in the dark, followed by the addition of a permeabilization fixation solution (BD Biosciences) for cell membrane permeabilization. Intracellular markers were then stained in the dark. The samples were analyzed using a flow cytometer (BD FACSCelesta, BD Biosciences), and the flow cytometry data were processed and analyzed using FlowJo 10.8.1.

### Activation of OT-I CD8^+^ T cells.

CD8^+^ T lymphocytes were meticulously isolated from the spleens and lymph nodes of mice using anti-CD8 magnetic beads (Miltenyi) in accordance with the manufacturer’s guidelines. These cells were subsequently activated with anti-CD3 antibody, anti-CD28 antibody, and IL-2. As an alternative approach, OT-I CD8^+^ T cells were cocultured with BM-derived DCs that had been pulsed with 10 μg/mL ovalbumin, as well as RM-1-OVA tumor cells, to simulate the antigen presentation process in a physiological context.

### CAR construction and lentiviral transduction.

Second-generation CAR incorporating the J591 single-chain variable fragment were constructed to target human PSMA. The CAR cassette comprised the J591 single-chain variable fragment linked to a CD8α hinge and transmembrane domain, followed by the 4-1BB costimulatory domain and the CD3ζ activation domain (BBζ configuration). A gene encoding a secreted form of IL-15 was included in the construct to support NK cell persistence. Third-generation baboon envelope-pseudotyped lentiviral vectors were produced by transfecting HEK293T cells with the transfer and packaging plasmids using Lipofectamine 3000 (Thermo Fisher Scientific) (56). Primary mouse NK cells, activated with IL-2 and IL-15, were transduced with the viral supernatant at a MOI of 5. CAR expression on transduced NK cells was confirmed by flow cytometry.

### Statistics.

All statistical analyses were performed using GraphPad Prism 9 software. Data are presented as the mean ± SEM, except for tumor growth curves, which are plotted as the mean ± SD to illustrate intra-group variability over time. Statistical significance was defined as *P* value < 0.05. Comparisons among multiple groups were analyzed by 1-way ANOVA, while comparisons between 2 groups were performed using Welch’s 2-tailed *t* tests. Survival curves were generated using the Kaplan-Meier method and compared with the log-rank test. Correlations between 2 continuous variables were assessed using Pearson’s correlation analysis. Longitudinal tumor growth data were evaluated by repeated-measure 2-way ANOVA.

### Study approval.

Animal experimental protocols were approved by the Institutional Animal Care and Use Committee of Tongji Hospital, Huazhong University of Science and Technology (ethical approval number TJH-202501003). PCa tissue specimens and peripheral blood serum of PCa patients were obtained from the Department of Urology, Tongji Hospital, Tongji Medical College, Huazhong University of Science and Technology. All patients signed informed consent forms. The collection methods of clinical specimens and the experimental uses were reviewed and approval was granted by the Medical Ethics Committee of Tongji Hospital, Tongji Medical College, Huazhong University of Science and Technology (ethical approval number TJ-IRB202503117).

### Data availability.

The RNA-seq data in raw FASTQ format have been deposited in the Genome Sequence Archive of the National Genomics Data Center, China National Center for Bioinformation (accession numbers CRA037192, CRA037195, and CRA037254; BioProject PRJCA056018) and are publicly accessible at https://ngdc.cncb.ac.cn/gsa Proteomics data are distributed under Open Archive for Miscellaneous Data of the National Genomics Data Center, China National Center for Bioinformation (accession numbers OMIX014438 and OMIX014439) and are publicly accessible at https://ngdc.cncb.ac.cn/omix All antibodies and reagents used in this study are presented in Supplemental Table 1. The primer sequences are available in Supplemental Table 2. All the unedited gels/blots are included in the supplemental materials. Values for all data points are presented in the Supporting Data Values file. Other methods are available in the Supplemental Methods.

## Author contributions

ZC, LL, and XH conducted most of the experiments and contributed equally to this work. ZC wrote the manuscript. LL and XH analyzed experimental data and the database. HP provided the experimental design and technical support. YW, CZ, and XG helped with the cell culture and data collection. P Liu, SM, and JZ assisted with the breeding and feeding of the mice. GQ and YP assisted with the collection of specimens. Z Wei, JL, and BL revised the manuscript. P Lan and Z Wang acquired funding and supervised the study.

## Funding support

- National Natural Science Foundation of China (823B2064, 82373333, 82271807, and 82471810).
- Hubei Province Science Fund for Distinguished Young Scholars (2022CFA057).
- Central Research Institute Fund of the Chinese Academy of Medical Sciences (2023-PT320-07).

## Supplementary Material

Supplemental data

Unedited blot and gel images

Supporting data values

## Version 1 01/27/2026

In-Press Preview

## Version 2 04/01/2026

Electronic publication
