# Supplementary material for: PARP inhibitors restore NK cell function via secretory crosstalk with tumor cells in prostate cancer
Source: J Clin Invest. 2026 Jan 27;136(7):e197157. doi: 10.1172/JCI197157 (PMC13038204; doi:10.1172/JCI197157)
Supplement: Supplemental data [file jci-136-197157-s312.pdf]

Supplemental Data

Supplemental Figure and Figure Legend

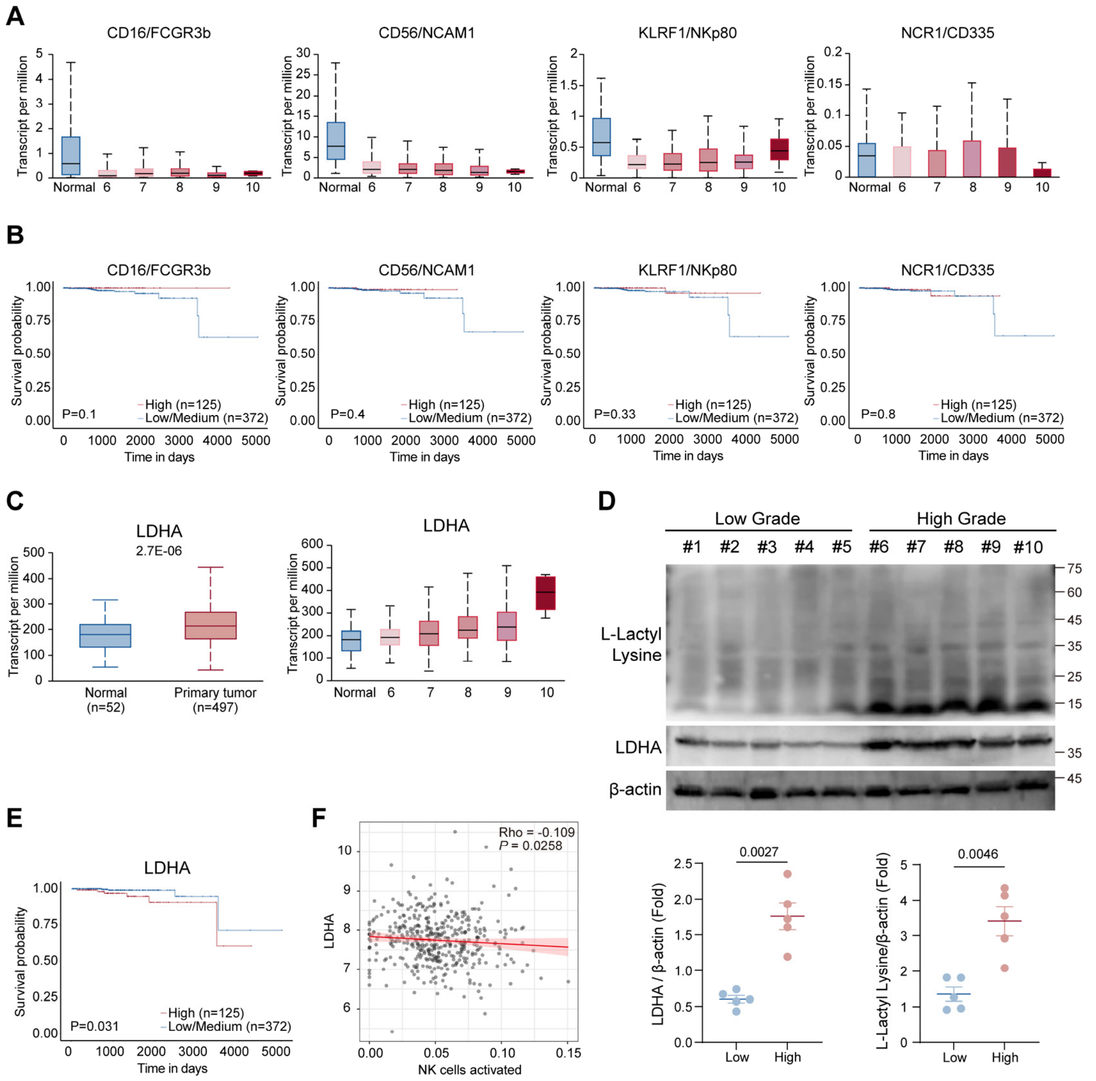

Supplemental Figure 1. NK cell infiltration and effector function improves prognosis in prostate cancer

(A) Expression levels of CD16, CD56, NCR1 (NKp46/CD335), and KLRF1 (NKp80) in normal tissues and prostate cancer with different Gleason scores analyzed using the TCGA database.

(B) Survival curves of prostate cancer patients stratified by high versus low expression of CD16, CD56, NCR1 (NKp46/CD335), and KLRF1 (NKp80) in the TCGA database.

(C) Differential expression of LDHA in normal tissues and prostate cancer with different Gleason scores analyzed using the TCGA database.

(D) Western blot analysis shows expression of LDHA and lysine lactylation in clinical tissue samples, with expression levels normalized to β-actin, N=5 per group. Data were analyzed by Welch's t-test.

(E) Survival curves of prostate cancer patients stratified by high versus low expression of LDHA in the TCGA database.

(F) Negative correlation between LDHA expression and activated NK cell infiltration in PCa patients from the TCGA database.

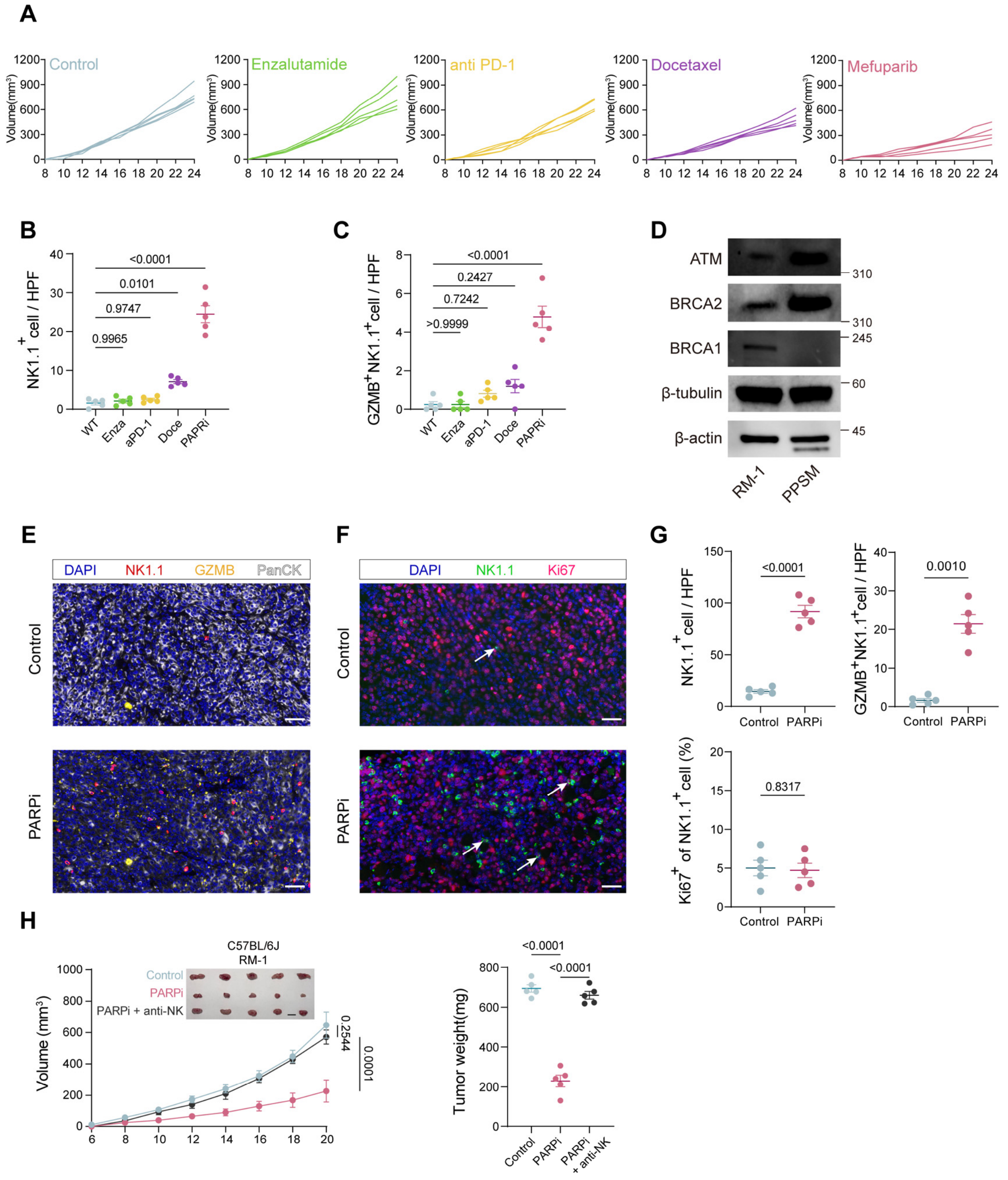

Supplemental Figure 2. PARPi treatment enhances NK cell infiltration in murine prostate cancer with intact HRR.

(A) Individual tumor growth curves of mice from (Figure 1D) treated with Enzalutamide, anti-PD-1, Docetaxel, or Mefuparib, N=5 per group.

(B and C) Quantification of average NK1.1$^+$ cells (B) and GZMB$^+$NK1.1$^+$ cells (C) per high-power field in immunofluorescence-stained tissues from (Figure 1F), and data are based on average counts from five random fields per sample, N=5 per group.

(D) Western blot analysis of BRCA1, BRCA2 and ATM expression in murine prostate cancer cell lines RM-1 and PPSM.

(E) Immunofluorescence staining of tumor tissues from (Figure 1G) treated with Mefuparib, showing DAPI (blue), NK1.1 (red), Granzyme B (yellow), and PanCK (white). Scale bar: 30 μm.

(F) Immunofluorescence staining of tumor tissues from (Figure 1G) treated with Mefuparib, showing DAPI (blue), NK1.1 (green) and Ki67 (violet). Scale bar: 30 μm.
(G) Statistical analysis of NK cell count, $GZMB^{+}$ NK cell count, and the proportion of $Ki67^{+}$ cells among NK cells. Data are based on average counts from five random fields per sample, N=5 per group.
(H) C57BL/6 mice were inoculated with RM-1 cells and then treated with PARPi in the setting of NK cell depletion or with NK cells intact. Tumor growth and weight were tracked, N=5 per group.
Tumor growth curves data were presented as mean ± SD, and analyzed by two-way ANOVA with Tukey's multiple comparisons test. Other data were presented as mean ± SEM. Data were analyzed by one-way ANOVA (B, C and H), and Welch's t-test (G).

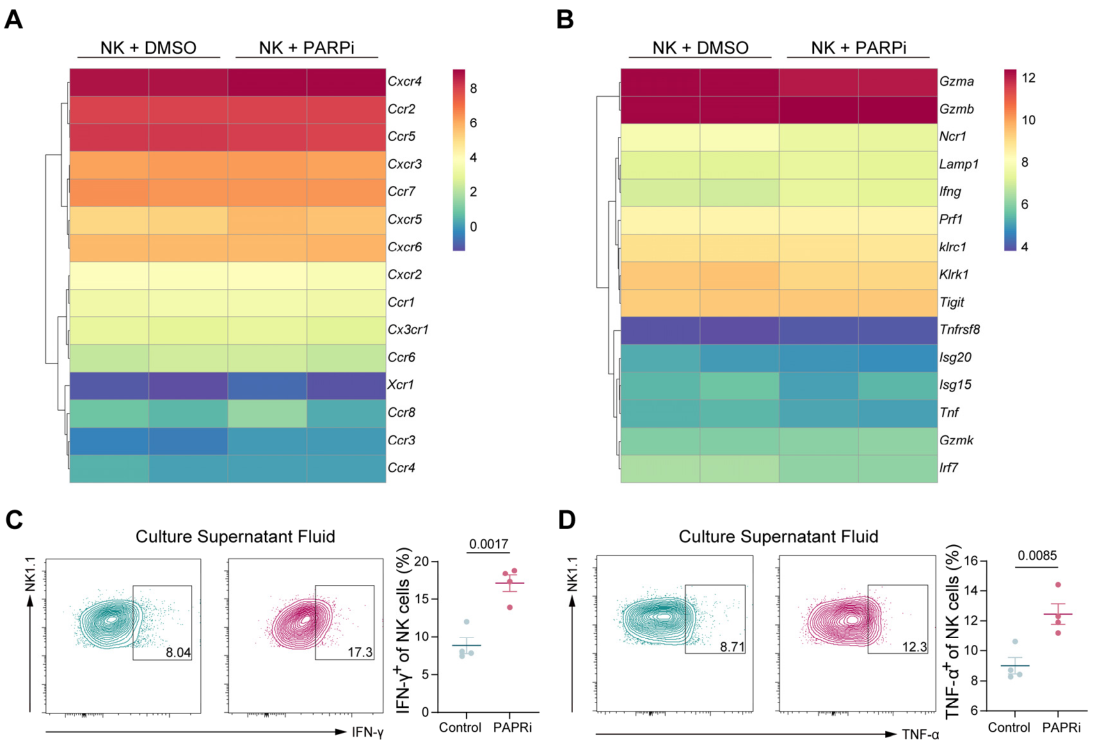

Supplemental Figure 3. PARPi does not directly alter NK cell chemotaxis or effector functions

(A and B) RNA sequencing analysis of chemotaxis-related (A) and effector function-related (B) gene expression changes in mouse NK cells treated with DMSO or PARPi (1 μM) for 24 hours.

(C and D) IFN-γ (C) and TNF-α (D) expression in mouse NK cells cultured with conditioned medium from RM-1 cells pre-treated with DMSO or PARPi (1 μM) for 24 hours, N=4 per group. Data were presented as mean ± SEM and analyzed by Welch's t-test.

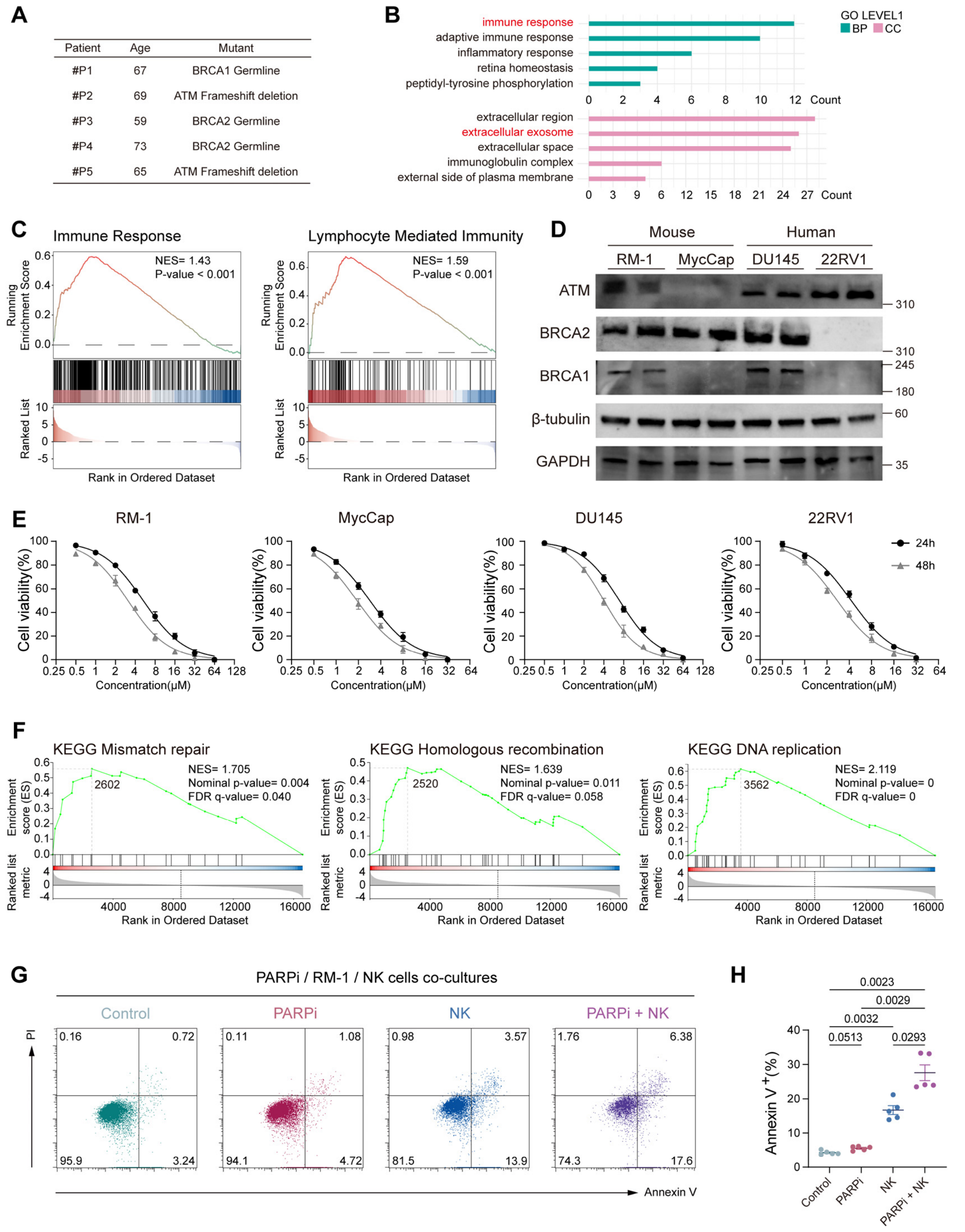

Supplemental Figure 4. PARP inhibitors induce active immune responses in prostate cancer

(A) Baseline characteristics of 5 enrolled patients.

(B) GO pathway alterations in serum proteins of patients post-PARPi treatment.

(C) Gene Set Enrichment Analysis showing enrichment of immune response and lymphocyte mediated immunity pathways.

(D) Western blot analysis of BRCA1, BRCA2 and ATM protein expression in RM-1, MycCap, DU145, and 22RV1 cells.

(E) IC_{50} values of Mefuparib in RM-1, MycCap, DU145, and 22RV1 cells.

(F) Pathway enrichment analysis of mismatch repair, homologous recombination, and DNA replication after PARPi treatment.
(G) Apoptosis of RM-1 prostate cancer cells co-cultured with DMSO control, PARPi (1 μM), NK cells (effector-to-target ratio 5:1), or PARPi (1 μM) + NK cells (E:T ratio 5:1) for 24 hours, assessed by Annexin V/PI staining.
(H) Quantification of RM-1 apoptosis across the four treatment groups, N=5 per group. Data were presented as mean ± SEM and analyzed by one-way ANOVA.

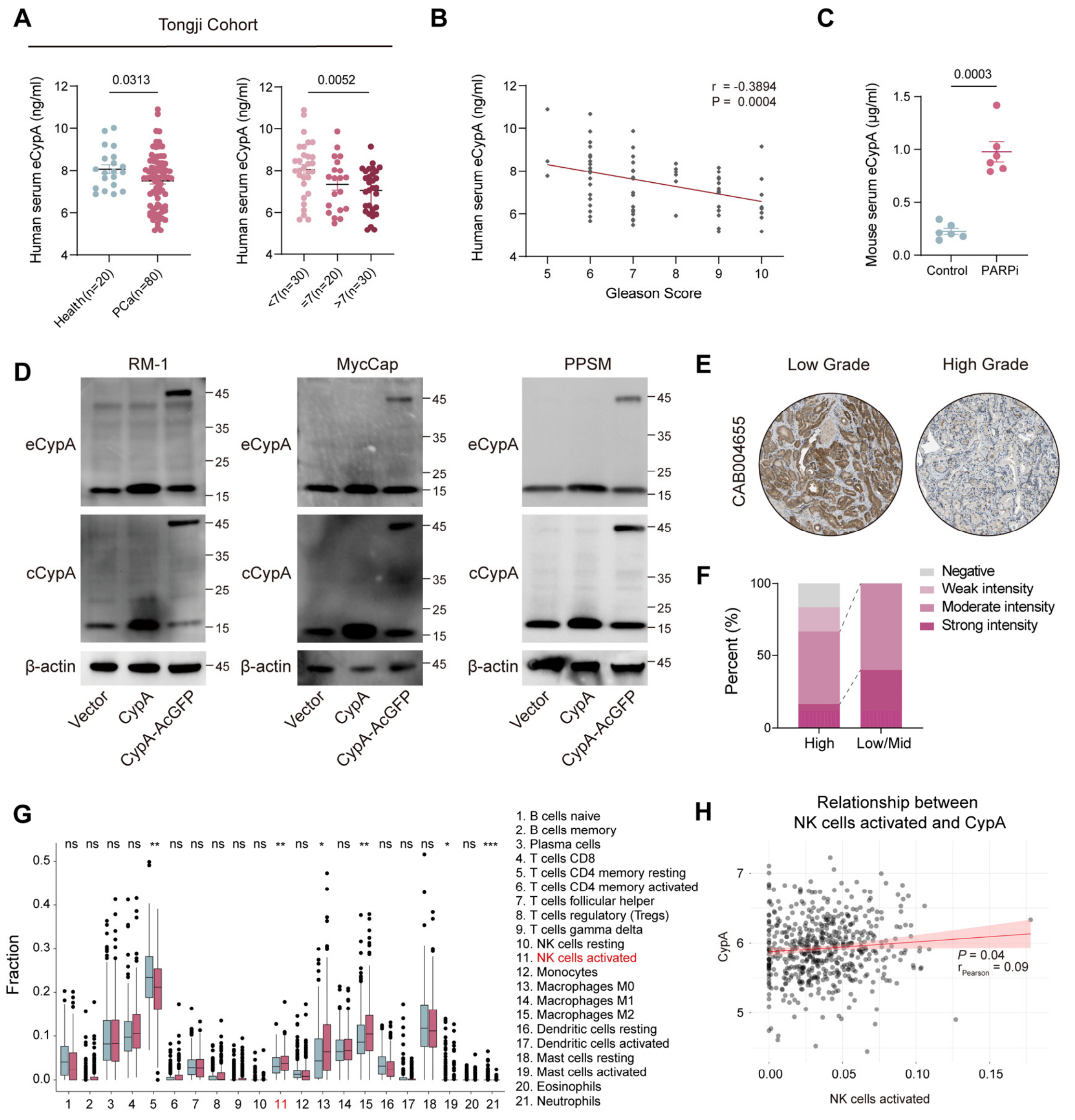

Supplemental Figure 5. CypA expression negatively correlates with PCa progression via eCypA secretion

(A) ELISA analysis of CypA levels in serum from healthy individuals (N=20) and prostate cancer patients (N=80) collected at Tongji Hospital.

(B) Correlation between serum CypA levels and Gleason scores in prostate cancer patients.

(C) Serum CypA levels in tumor-bearing mice from (Figure 1I) treated with or without PARPi, N=6 per group.

(D) Western blot analysis of intracellular and extracellular CypA levels in PCa cells with or without CypA plasmia.

(E and F) CypA expression in high-grade versus low-grade PCa samples from the Protein Atlas database (CAB004655) (E) and quantification of CypA levels (F).

(G) Immune infiltration analysis based on CypA expression subgroups in PCa patients from the TCGA database.

(H) Positive correlation between CypA expression and activated NK cell infiltration in PCa patients from the TCGA database.

Data were presented as mean ± SEM and analyzed by Welch's t-test.

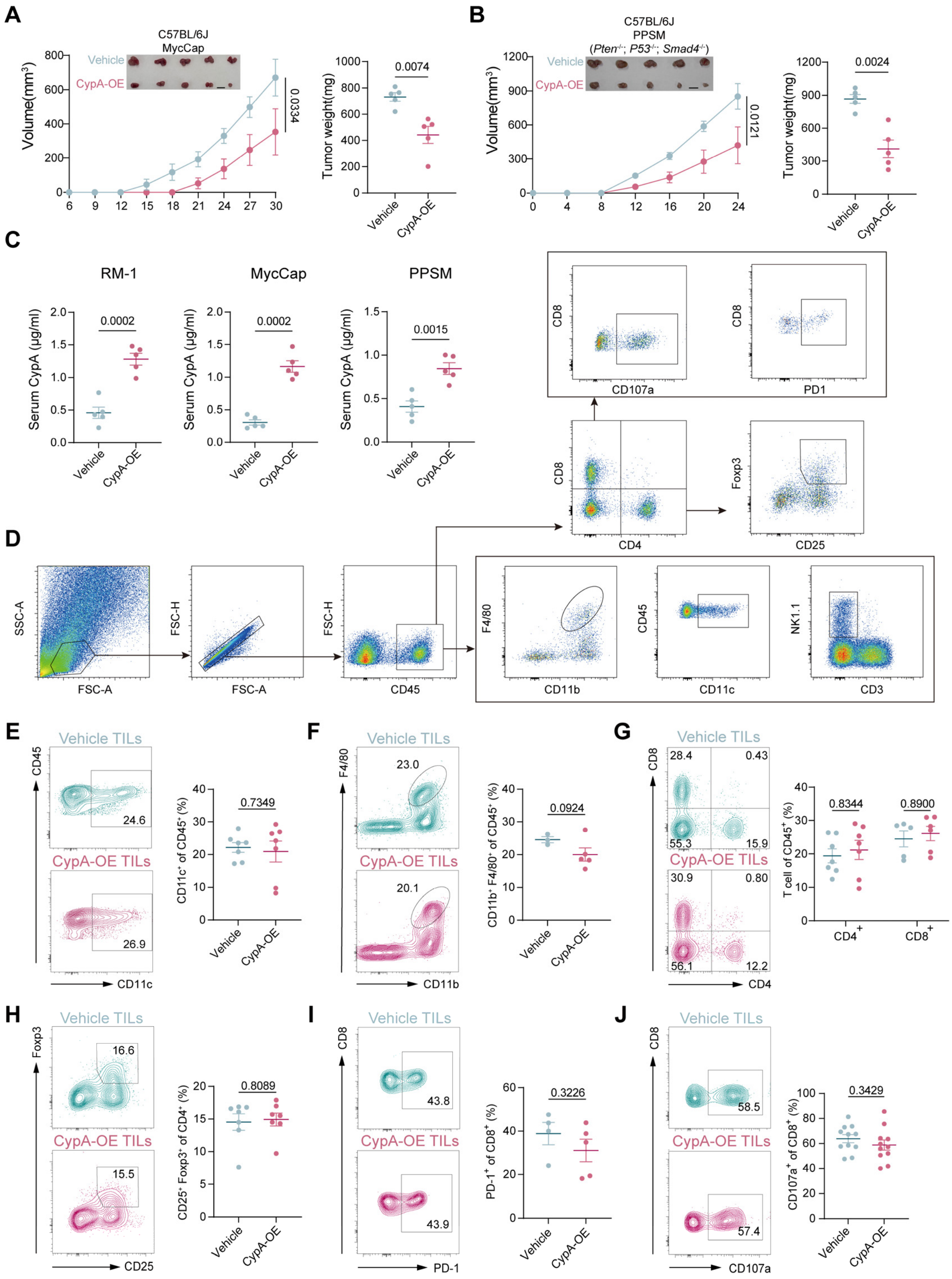

Supplemental Figure 6. CypA-OE does not alter other immune cell populations in the PCa microenvironment

(A and B) Tumor growth curves and weight in C57BL/6 mice inoculated with MycCap (A) and PPSM (B) (Vehicle/CypA-OE) cell lines, N=5 per group.

(C) Serum CypA levels in mice inoculated with CypA-OE PCa cells, N=5 per group.

(D) Gating strategy for TILs analyzed by flow cytometry.

(E-H) Proportions of DCs (E), macrophages (F), $CD4^+$ T cells, $CD8^+$ T cells (G), and Tregs (H) in the TME of CypA-OE prostate cancer, with quantification panels.

(I and J) PD-1 expression (I) and CD107a expression (J) in $CD8^+$ T cells within the TME of CypA-OE prostate cancer, with quantification panels.
Tumor growth curves data were presented as mean ± SD, and analyzed by two-way ANOVA with Tukey's multiple comparisons test. Other data were presented as mean ± SEM and analyzed by Welch's t-test.

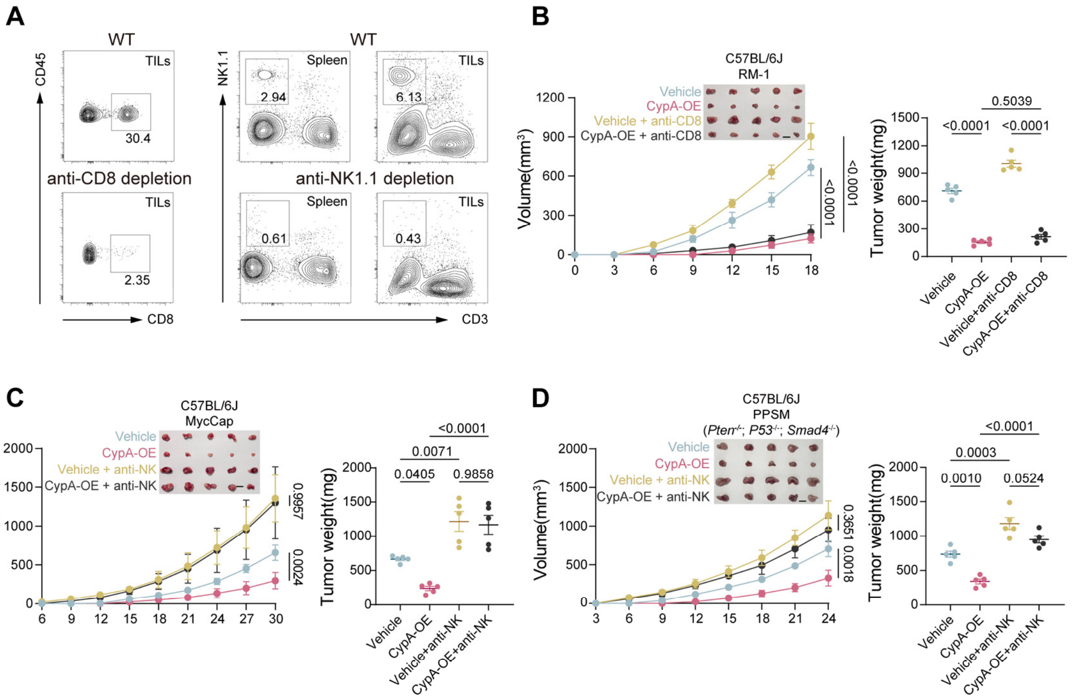

Supplemental Figure 7. The anti-cancer effect of CypA overexpression depends on NK cells

(A) Validation of depletion efficiency using $CD8^+$ T cell-depleting antibodies and NK cell-depleting antibodies via flow cytometry.

(B) Tumor growth curves and weights in $CD8^+$ T cell-depleted C57BL/6 mice inoculated with CypA-OE tumor cells, N=5 per group.

(C and D) Tumor growth curves and weights in NK cell-depleted C57BL/6 mice inoculated with MycCap (C) and PPSM (D) (Vehicle/CypA-OE) cell lines, N=5 per group.

Tumor growth curves data were presented as mean ± SD, and analyzed by two-way ANOVA with Tukey's multiple comparisons test. Tumor weights data were presented as mean ± SEM, and analyzed by one-way ANOVA.

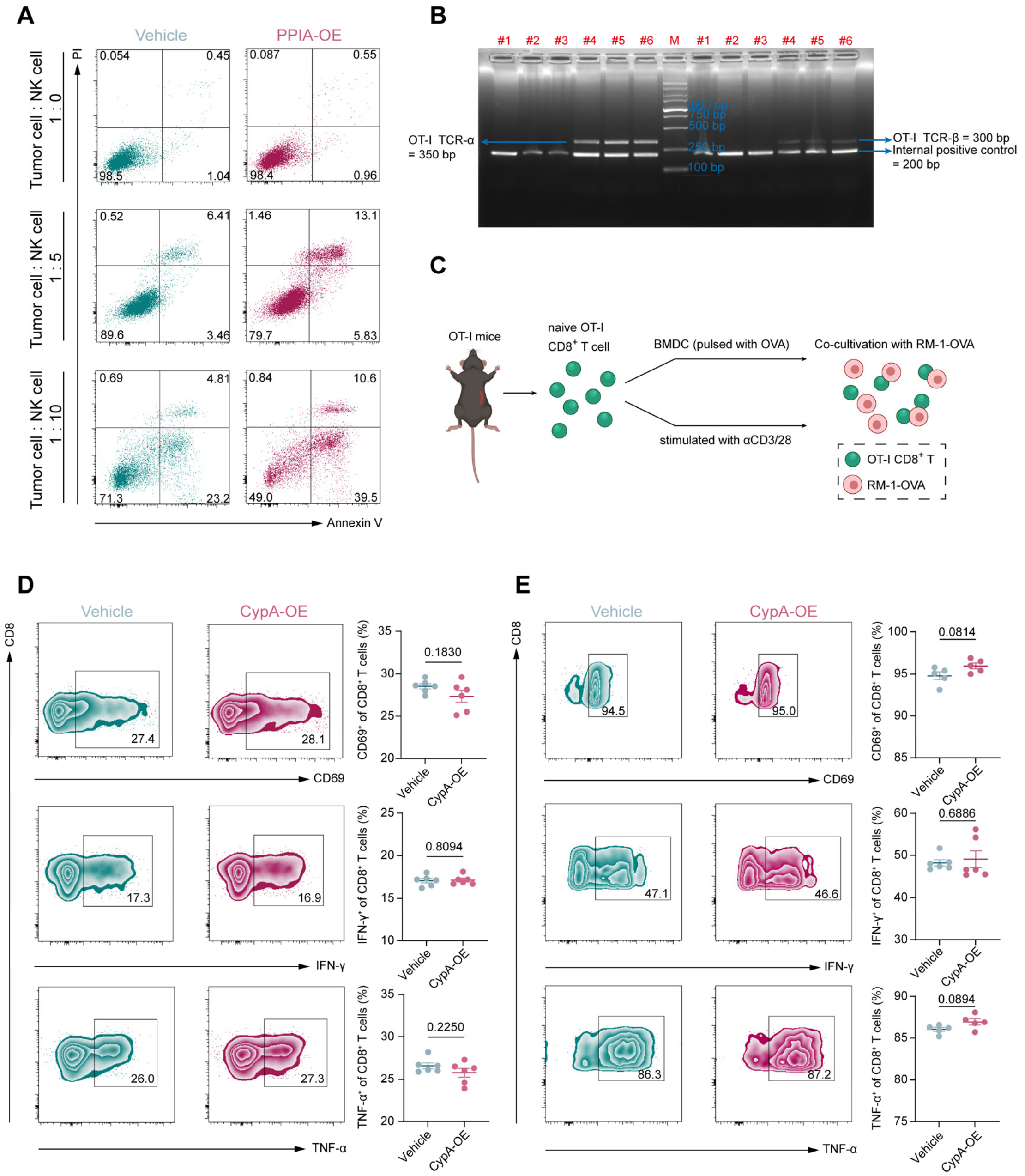

Supplemental Figure 8. CypA enhances NK cell effector functions but not CD8+ T cells

(A) Apoptosis of RM-1 cells (transfected with CypA-OE plasmid or vehicle control) co-cultured with mouse NK cells at ratios of 1:0, 1:5, and 1:10 for 24 hours, assessed by Annexin V/PI staining.

(B) Genotyping of OT- I mice was performed by DNA agarose gel electrophoresis.

(C) Schematic diagram of the experimental co-culture. Naive CD8+ T cells from OT-1 mice were activated via one of two strategies: using OVA-pulsed BMDCs or through anti-CD3/CD28 stimulation, and then co-cultured with RM-1-OVA cells.

(D) Flow cytometry analysis of CD69, IFN-γ, and TNF-α expression in OT-I CD8+ T cells (stimulated by BMDCs) co-cultured with RM-1-OVA cells transfected with CypA-OE or vehicle control, N=6 per group.

(E) Flow cytometry analysis of CD69, IFN-γ, and TNF-α expression in OT-I CD8$^+$ T cells (stimulated by anti-CD3/CD28) co-cultured with RM-1-OVA cells transfected with CypA-OE or vehicle control, N=5 per group.
Data were presented as mean ± SEM and analyzed by Welch's t-test.

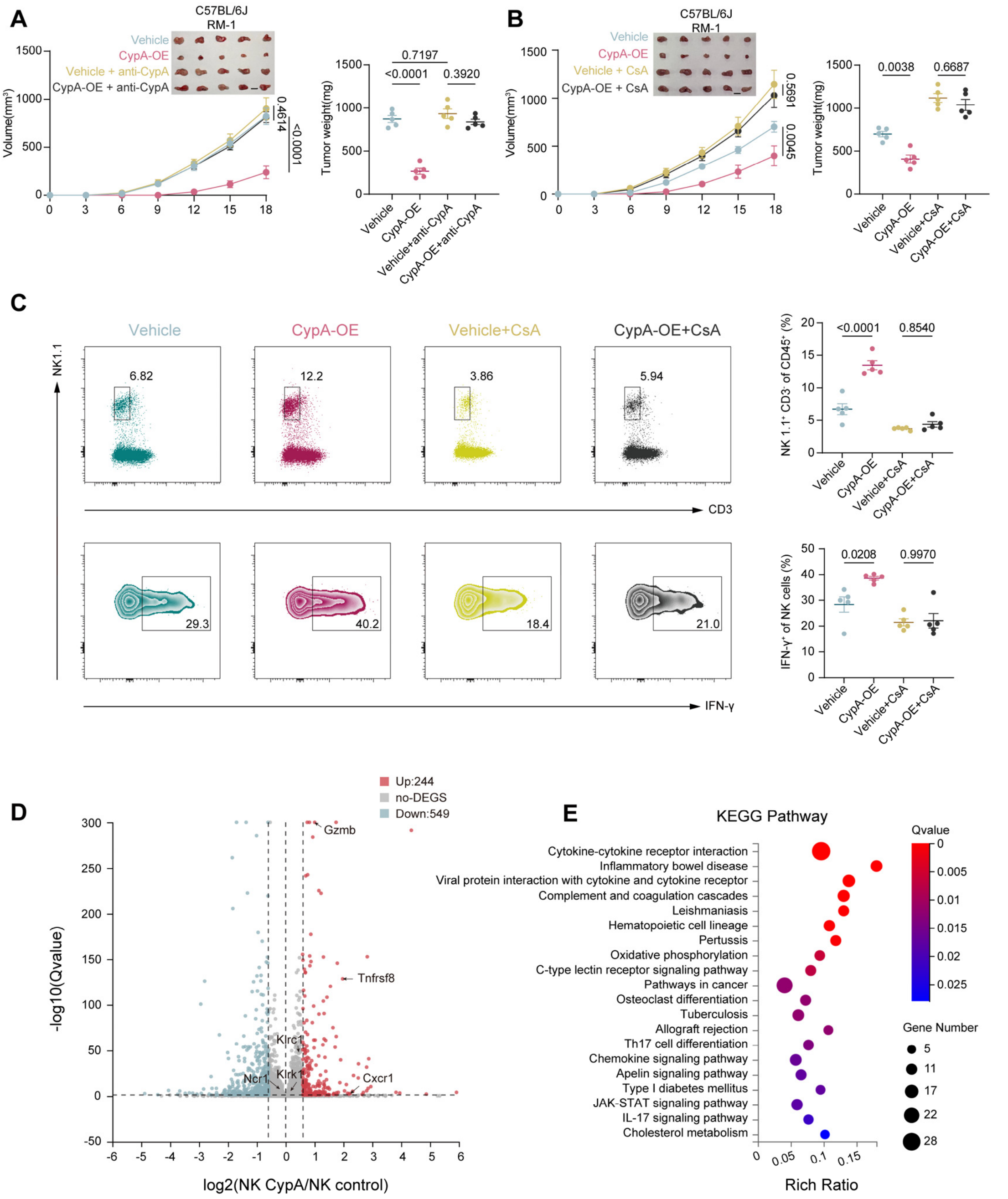

Supplemental Figure 9. The tumor-suppressive effect was abrogated upon eCypA blockade

(A) Tumor growth curves and weights in mice bearing RM-1-Vehicle or RM-1-CypA-OE tumors, treated with or without anti-CypA, N=5 per group.

(B) Tumor growth curves and weights in mice bearing RM-1-Vehicle or RM-1-CypA-OE tumors, treated with or without CsA, N=5 per group.

(C) Flow cytometry analysis of TINKs proportions and IFN-γ expression from (Supplemental Figure 9B).

(D) Volcano plot showing differential gene expression in NK cells treated with CypA.

(E) KEGG pathway enrichment analysis reveals the top 20 enriched pathways.

Tumor growth curves data were presented as mean ± SD, and analyzed by two-way ANOVA with Tukey's multiple comparisons test. Other data were presented as mean ± SEM and analyzed by one-way ANOVA.

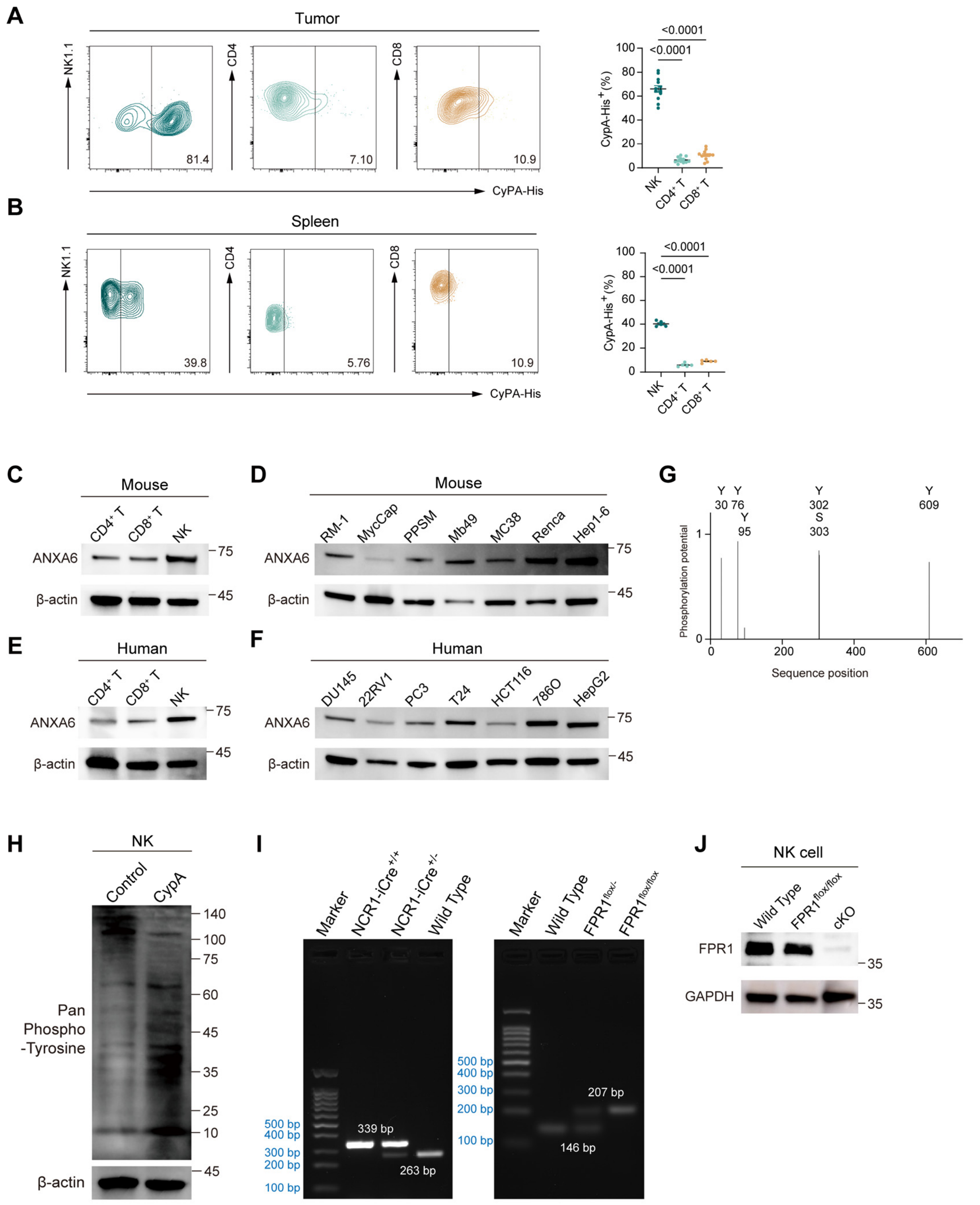

Supplemental Figure 10. ANXA6 expression in immune and tumor cells

(A) Flow cytometric analysis of the binding proportion of His-tagged recombinant protein to NK, $CD4^+$, or $CD8^+$ T cells in the tumor microenvironment, N=12 per group.
(B) Flow cytometric analysis of the binding proportion of His-tagged recombinant protein to NK, $CD4^+$, or $CD8^+$ T cells in the mouse spleen, N=5 per group.
(C and D) Western blot analysis of ANXA6 expression in murine immune cells (NK, $CD4^+$, $CD8^+$ T cells) (C) and murine tumor cell lines: prostate cancer (RM-1, MycCap, PPSM), bladder cancer (MB49), colorectal cancer (MC38), renal cancer (Renca), and hepatocellular carcinoma (Hep1-6) (D).
(E and F). Western blot analysis of ANXA6 expression in human immune cells (NK, $CD4^+$, $CD8^+$ T cells) (E) and human tumor cell lines: prostate cancer (DU145, 22RV1,

PC3), bladder cancer (T24), colorectal cancer (HCT116), renal cancer (786-O), and hepatocellular carcinoma (HepG2) (F).

(G) Analysis using NetPhos, UniProt, and PhosphoSitePlus revealed high-confidence phosphorylation sites on the ANXA6 protein.

(H) Global phosphorylation level changes in NK cells after CypA stimulation.

(I) Genotyping of *Ncr1-iCre* mice and *Fpr1*$^{flox/flox}$ mice were performed by DNA agarose gel electrophoresis.

(J) Western blot analysis demonstrating FPR1 protein expression in NK cells isolated from wild-type, *Fpr1*$^{flox/flox}$, and *Ncr1-iCre+-Fpr1*$^{flox/flox}$ (cKO) mice.

Data were presented as mean ± SEM and analyzed by one-way ANOVA.

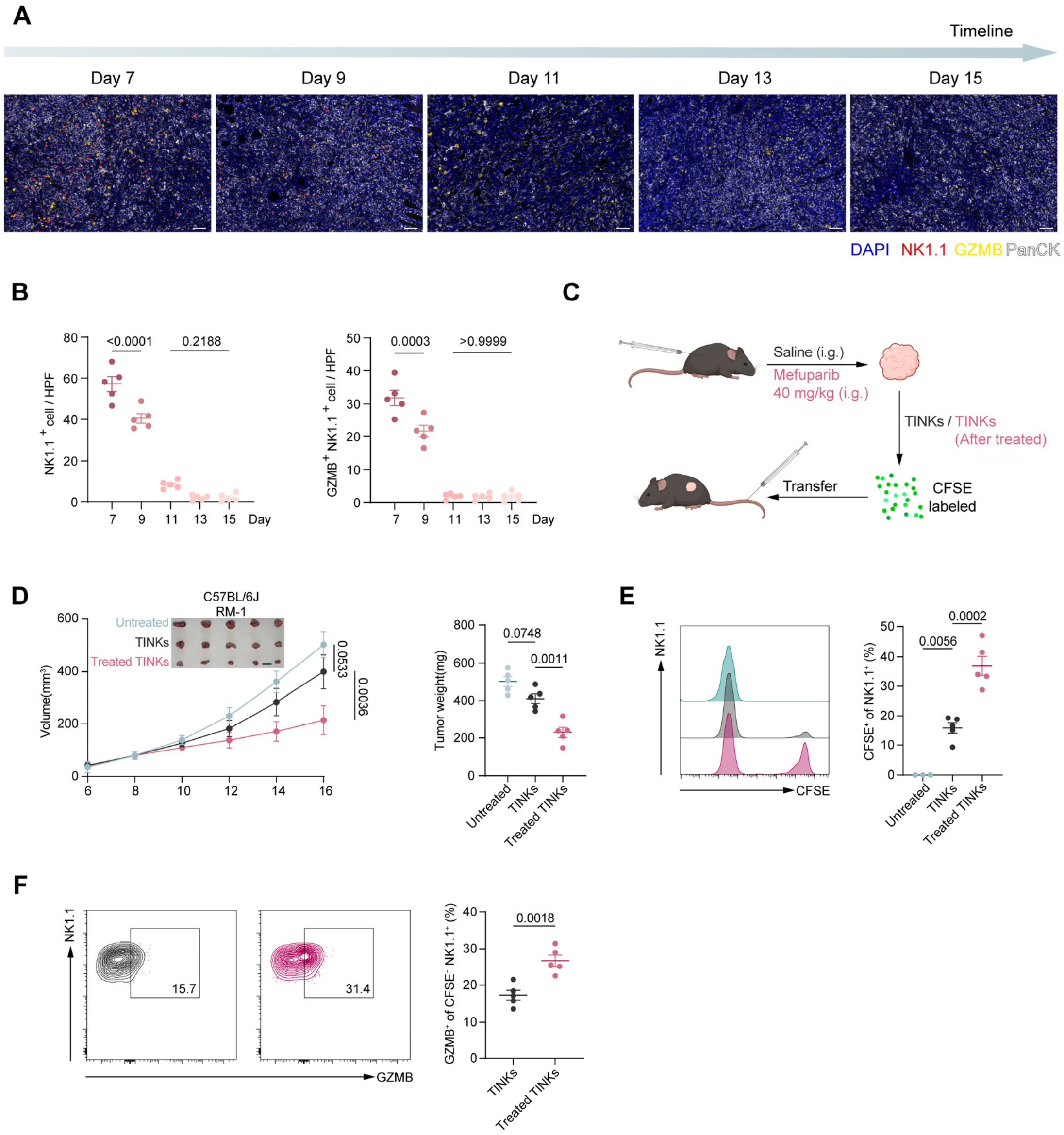

Supplemental Figure 11. Rapid functional exhaustion of NK cells in the PCa tumor microenvironment

(A) Immunofluorescence staining showing NK cell and GZMB$^+$NK cell infiltration in RM-1 prostate tumors at days 7, 9, 11, 13, and 15 post-inoculation. DAPI (blue), NK1.1 (red), Granzyme B (yellow), PanCK (white). Scale bar: 50 µm.

(B) Quantification of average NK1.1$^+$ cells and GZMB$^+$NK1.1$^+$ cells per high-power field in Supplemental Figure 10A. Data are based on average counts from five random fields per sample, N=5 per group.

(C) CFSE-labeled TINKs isolated from 7-week-old C57BL/6 mice bearing prostate tumors (treated with PARPi or saline control) were adoptively transferred via tail vein into tumor-bearing recipient mice.

(D) Tumor growth curves and weights in recipient mice receiving no treatment, adoptive transfer of untreated TINKs, or PARPi-treated TINKs, N=5 per group.

(E) Flow cytometry analysis of total NK cell proportions and CFSE-labeled NK cell proportions in the tumor microenvironment of mice from (Figure S8C), N=5 per group.

(F) Granzyme B expression in CFSE-labeled NK cells, N=5 per group.

Tumor growth curves data were presented as mean ± SD, and analyzed by two-way ANOVA with Tukey's multiple comparisons test. Other data were presented as mean ± SEM. Data were analyzed by one-way ANOVA (B, D and E), and Welch’s t-test (F).

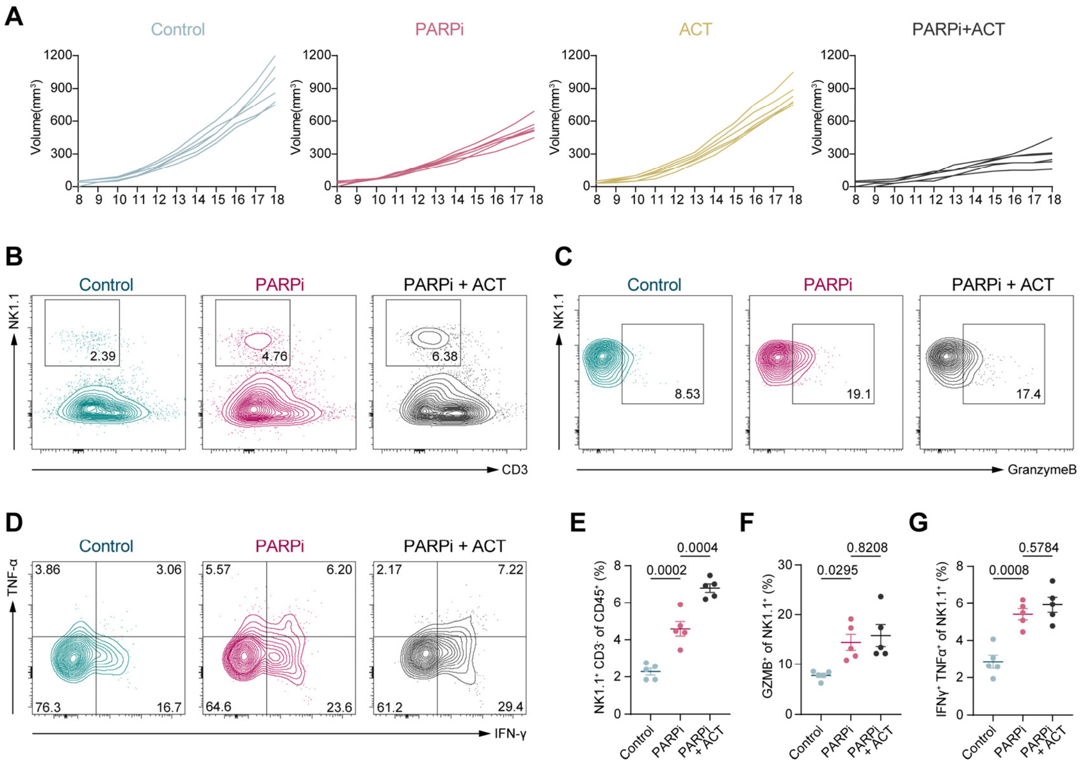

Supplemental Figure 12. Adoptive cell therapy augments PARPi-induced anti-tumor immunity

(A) Individual tumor growth curves of mice from (Figure 7B) across treatment groups, N=6 per group.

(B-G) Flow cytometry analysis of tumor-infiltrating lymphocytes: NK cell proportions (B), GZMB⁺ (C), and IFN-γ⁺ (D) levels, with quantification panels (E-G). N=5 per group.

Data were presented as mean ± SEM and analyzed by one-way ANOVA.

Supplemental Table1

The antibodies and reagents used in this study

Name	Vendor	Catalog
**Antibodies**		
InVivoMAb anti-mouse CD8α	Bioxcell	BE0061
InVivoMAb anti-mouse NK1.1	Bioxcell	BE0036
InVivoMAb anti-mouse CD3ε	Bioxcell	BE0001
InVivoMAb anti-mouse CD28	Bioxcell	BE0015
NCR1 Rabbit mAb	Zenbio	R51204
NK1.1/CD161 Rabbit mAb	Cell Signaling Technology	39197
Anti-Granzyme B mAb	Abcam	ab255598
Pan-CK pAb	AiFang biological	AF20164
Ki67 Rabbit mAb	Abclonal	A20018
GAPDH Monoclonal antibody	Proteintech	60004-1-Ig
Beta Actin Monoclonal antibody	Proteintech	66009-1-Ig
Beta Tubulin Rabbit mAb	Abclonal	A12289
Cyclophilin A Rabbit pAb	Zenbio	122908
LDHA Rabbit mAb	Zenbio	R24822
Anti-L-Lactyl Lysine Rabbit mAb	PTMBio	PTM-1401RM
BRCA1 Rabbit pAb	Abclonal	A11034
BRCA2 Rabbit pAb	Abclonal	A2435
ATM Rabbit mAb	Abclonal	A19650
Annexin VI Polyclonal antibody	Proteintech	12542-1-AP
6*His, His-Tag Monoclonal antibody	Proteintech	66005-1-Ig
Pan Phospho-Tyrosine Rabbit mAb	Abclonal	AP1162
FPR1 Rabbit Polyclonal Antibody	HUABIO	R1511-35
ERK1/2 Rabbit mAb	Abclonal	A4782
Phospho-ERK1-T202/Y204 + ERK2-T185/Y187 Rabbit mAb	Abclonal	AP0974
Pan-Akt Rabbit pAb	Abclonal	A18120
Phospho-Akt (Ser473) Rabbit Monoclonal Antibody	Cell Signaling Technology	4060
mTOR Rabbit pAb	Abclonal	A11355
Phospho-mTOR (Ser2448) Rabbit mAb	Cell Signaling Technology	5536
PSMA/FOLH1 Rabbit mAb	Abclonal	A9547
Brilliant Violet 785 anti-mouse CD45 Antibody	BioLegend	103149
PerCP anti-mouse CD3 Antibody	BioLegend	100287
APC anti-mouse NK-1.1 Antibody	BioLegend	156505
FITC anti-human/mouse Granzyme B Antibody	BioLegend	515403
PE anti-mouse IFN-γ Antibody	BioLegend	505807
PE/Cyanine7 anti-mouse TNF-α Antibody	BioLegend	506323

PE Rat Anti-Mouse CD181 (CXCR1)	BD Biosciences	566383
Pacific Blue anti-mouse CD4	BioLegend	100427
APC/Cyanine7 anti-mouse CD8a Antibody	BioLegend	100713
Brilliant Violet 421 anti-mouse CD25 Antibody	BioLegend	113705
PE anti-mouse FOXP3 Antibody	BioLegend	126403
FITC anti-mouse CD279 (PD-1) Antibody	BioLegend	135213
FITC anti-mouse/human CD11b Antibody	BioLegend	101205
APC anti-mouse F4/80 Antibody	BioLegend	123115
PE/Cyanine7 anti-mouse CD11c Antibody	BioLegend	117317
APC anti-mouse CD69 Antibody	BioLegend	104513
**Chemicals, peptides, and recombinant proteins**		
CFSE	BD Biosciences	565082
DAPI	Thermo Fisher Scientific	D3571
TRIzol	Thermo Fisher Scientific	A33251
Anti-APC MicroBeads	Miltenyi Biotec	130-090-855
DMSO	Sigma	D2065
1640 medium	Gibco	8122012
DMEM	Gibco	8122122
Advanced DMEM	Gibco	12491015
Fetal Bovine Serum	Gibco	10099
Lipofectamine 3000	Thermo Fisher Scientific	L3000-008
Ovalbumin	Genescript	RP10611CN
Glutamax	Gibco	35050061
HEPES	Gibco	15630080
Noggin	MCE	HY-P700286
R-spondin	MCE	HY-P78344
B-27	Gibco	17504044
EGF	Novoprotein	CH28
Dihydrotestosterone	Selleck	S4757
A8301	Selleck	S7692
Recombinant Mouse IL-2	Abclonal	RP01384
Recombinant Mouse IL-15	Abclonal	RP01676
Recombinant Mouse CypA Protein	Abclonal	RP01609LQ
Mefuparib	MCE	HY-122661
Enzalutamide	MCE	HY-70002
Docetaxel	MCE	HY-B0011
D-Luciferin, Potassium Salt	Yeasen	40902ES
Human CypA (Cyclophilin A) ELISA Kit	Elabscience	E-EL-H6096
Mouse Cyclophilin A (CyPA) ELISA Kit	Abclonal	RK02732
DCFH-DA	MCE	HY-D0940

Brefeldin A	beyotime	S1536
Annexin V-APC/PI Apoptosis Kit	Multi Sciences	AT107
Cell Counting Kit-8	beyotime	C0037
Mitochondrial membrane potential assay kit with JC-1	beyotime	C2006
Cyclosporin A	MCE	HY-B0579
HCH6-1	MCE	HY-101283
Mk-2206	MCE	HY-108232
SCH772984	MCE	HY-50846

Supplemental Table2

List of primers used in this study

**Primers and sequences for plasmid construction**	
pX459-Mus BRCA1 sg-F	GGCGTCGATCATCCAGAGCG
pX459-Mus BRCA1 sg-R	CGCTCTGGATGATCGACGCC
pLVX-CMV-Mus CypA-F	CGGATCGGGTTTAAACGGATCCGCCACCATG GTCAACCCCACCGTGTT
pLVX-CMV-Mus CypA-R	GGGCCCTCTAGACTCGAGCGGCCGCTCGAGC TGTCCACAGTCGGAAA
pLVX-CMV-Mus CypA-linker-AcGFP-F	GAATTCGCCACCATGGTCAACCCCACCGTGT T
pLVX-CMV-Mus CypA-linker-AcGFP-R	TCCACTCCCTCCGCCGCCGGATCCGAGCTGT CCACAGTCGGAAA
**Primers and sequences for mouse genotyping**	
OT-I mice-Tcrα-F	CAGCAGCAGGTGAGACAAAGT
OT-I mice-Tcrα-R	GGCTTTATAATTAGCTTGGTCC
OT-I mice-Tcrβ-F	AAGGTGGAGAGAGACAAAGGATTC
OT-I mice-Tcrβ-R	TTGAGAGCTGTCTCC
OT-I mice-Internal Positive Control-F	CAAATGTTGCTTGTCTGGTG
OT-I mice-Internal Positive Control-R	GTCAGTCGAGTGCACAGTTT
Ncr1-iCre-F1	GTGTCCATCCCTGAAATCATGCAG
Ncr1-iCre-F2	CTATGATGGAACTGAAGGCAACTC
Ncr1-iCre-R	GGCCATCACATTGGAACGAAG
Fpr1-flox-F	TCATCCTTTCAGAAACCTCTGTCT
Fpr1-flox-R	CTCAGAACTACTTTCCAAGCCAAG

Supplemental Methods

Elisa Assays.

The concentration of CypA in human serum, mouse serum, and cell culture supernatant was quantified using a commercial sandwich ELISA kit. Briefly, samples and serially diluted standards were added to the pre-coated plate and incubated. Following washes, a biotinylated detection antibody, then streptavidin-HRP, and finally TMB substrate were sequentially added with incubation and wash steps between each. The reaction was stopped, and the absorbance at 450 nm was measured. Sample concentrations were calculated by interpolation from the standard curve.

Immunofluorescence

Paraffin-embedded tissue sections (4 μm) from mouse or human prostate tumors were prepared for immunofluorescence analysis. Briefly, tissues were fixed in 4% paraformaldehyde, dehydrated, embedded in paraffin, and sectioned. After deparaffinization and rehydration, antigen retrieval was performed by heating the slides in retrieval buffer at 95°C for 20 minutes. Sections were permeabilized with 0.5% Triton X-100, blocked with 10% normal goat serum, and incubated with primary antibodies overnight at 4°C. Following PBS washes, sections were incubated with species-matched fluorophore-conjugated secondary antibodies for 1 hour at room temperature protected from light. Nuclei were counterstained with DAPI, and slides were mounted with anti-fade mounting medium. Imaging was performed by scanning the slides with a 3DHISTECH's Slide Converter.

Transmission Electron Microscopy

Mouse NK cells were fixed with 2.5% glutaraldehyde at 4°C for 12 h, followed by secondary fixation in 2% osmium tetroxide to enhance contrast of ultrastructural details. After washing, samples were stained with 1% aqueous uranyl acetate. Dehydration was performed through a graded ethanol series (50%, 70%, 90%, 95%, and 100%), and the samples were embedded in Eponate 12 resin. Ultrathin sections were cut, placed on copper grids, and stained with lead citrate and uranyl acetate. Sections were examined using a Tecnai G2 Spirit transmission electron microscope (FEI/Thermo Fisher Scientific) operated at 120 kV.
