# Supplementary material for: PARP inhibitors restore NK cell function via secretory crosstalk with tumor cells in prostate cancer
Source: J Clin Invest. 2026 Jan 27;136(7):e197157. doi: 10.1172/JCI197157 (PMC13038204; doi:10.1172/JCI197157)

Original Blots and Gels

Figure 2E

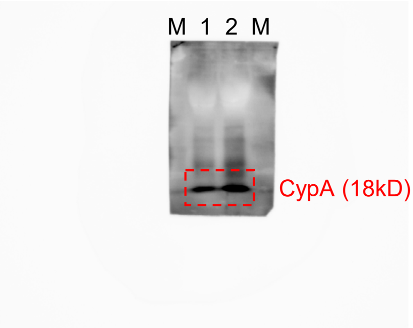

M:180kD protein marker
1:Myc-Cap control (supernatant)
2:Myc-Cap PARPi (supernatant)

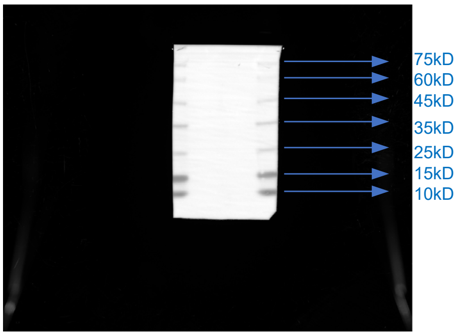

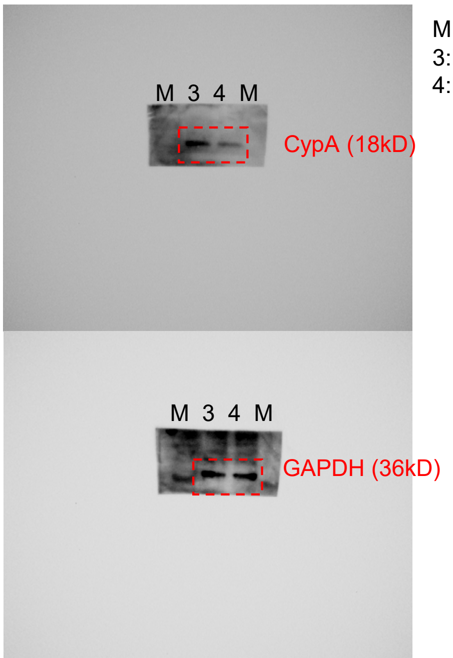

M:180kD protein marker
3:Myc-Cap control
4:Myc-Cap PARPi

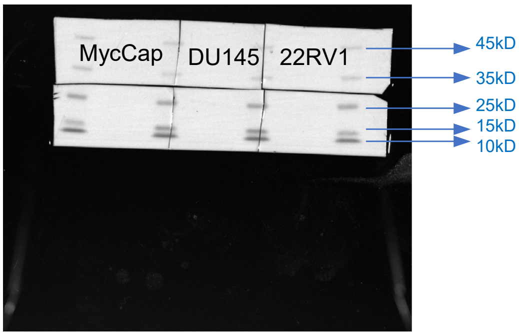

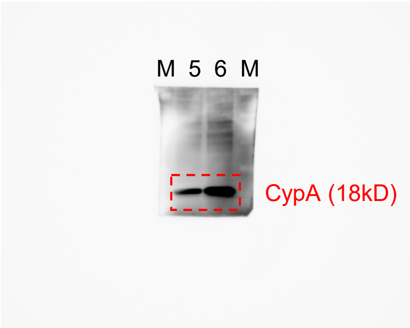

M:180kD protein marker
5:RM-1 control (supernatant)
6:RM-1 PARPi (supernatant)

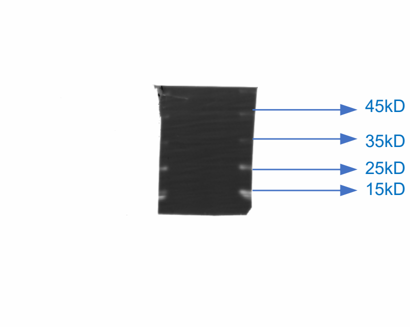

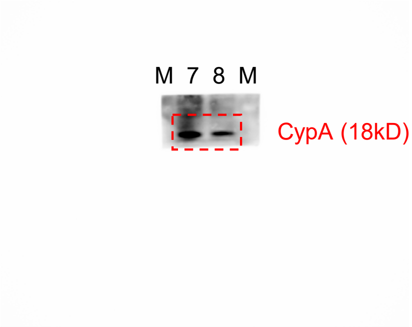

M:180kD protein marker
7:RM-1 control
8:RM-1 PARPi

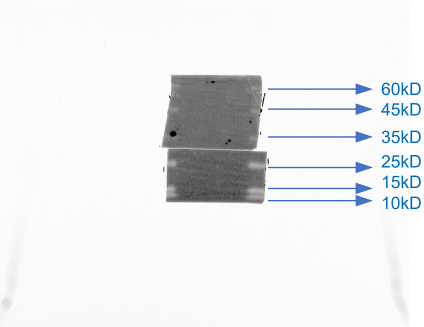

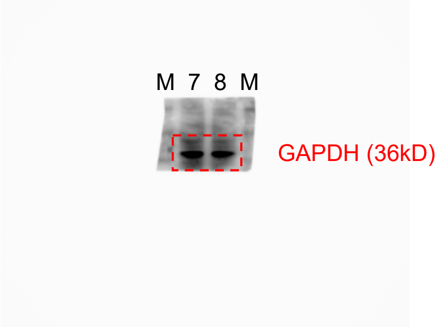

Figure 2F

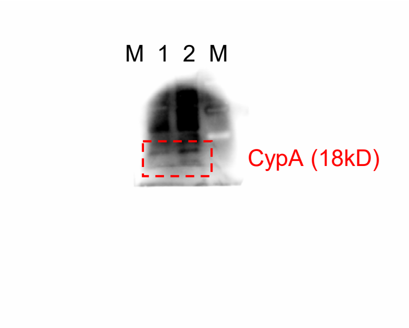

M:180kD protein marker
1:DU145 control (supernatant)
2:DU145 PARPi (supernatant)

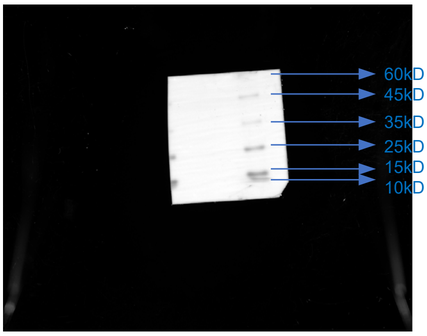

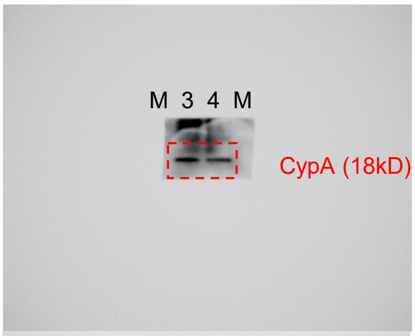

M:180kD protein marker
3:DU145 control
4:DU145 PARPi

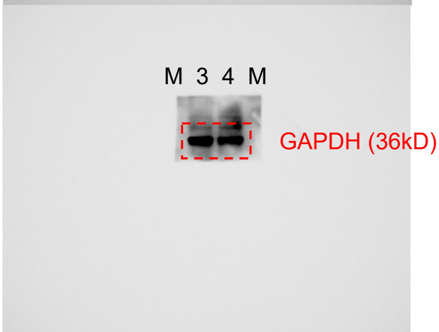

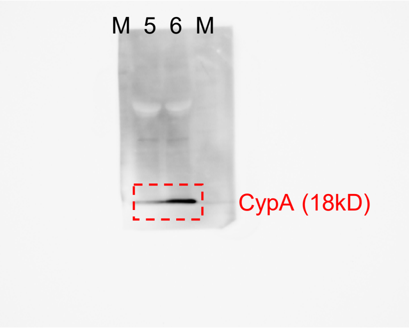

M:180kD protein marker
5:22RV1 control (supernatant)
6:22RV1 PARPi (supernatant)

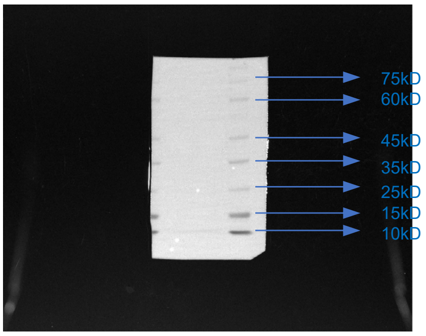

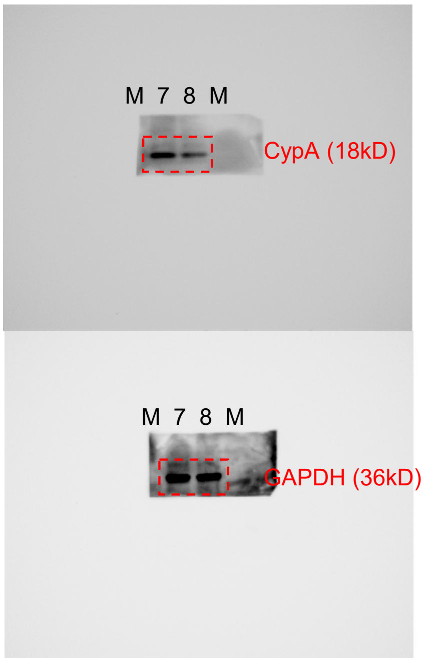

M:180kD protein marker
7:22RV1 control
8:22RV1 PARPi

Figure 2L

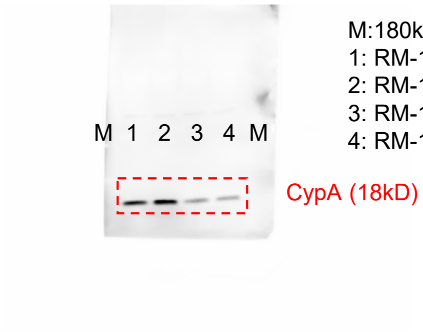

M:180kD protein marker
1: RM-1 Control (supernatant)
2: RM-1 PARPi (supernatant)
3: RM-1 BFA (supernatant)
4: RM-1 PARPi, BFA (supernatant)

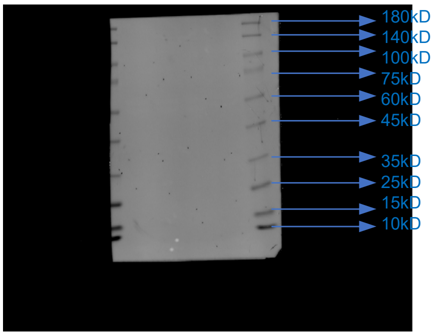

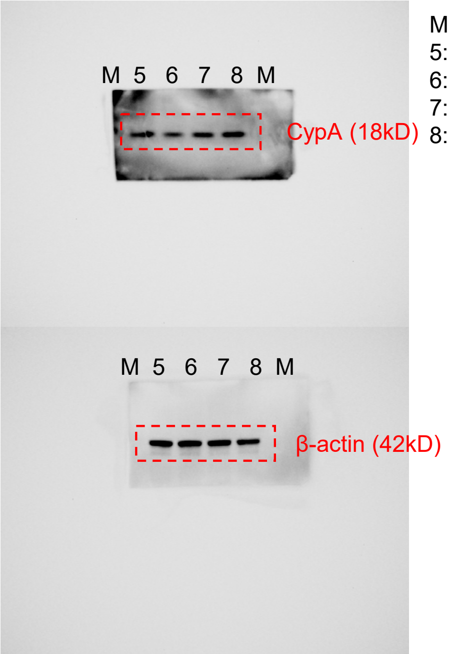

M:180kD protein marker
5: RM-1 Control
6: RM-1 PARPi
7: RM-1 BFA
8: RM-1 PARPi, BFA

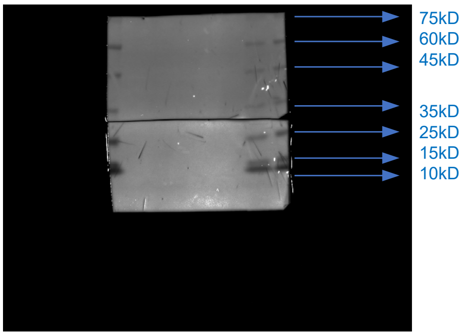

Figure 5E

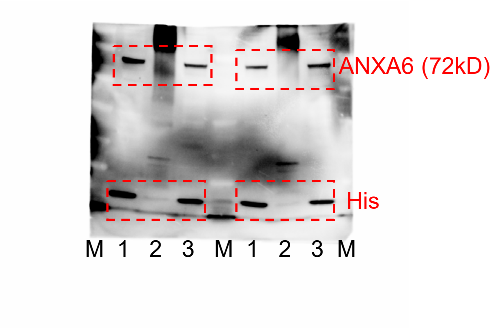

M:180kD protein marker
1: NK cell input
2: IgG
3: NK cell His-mAb (IP)
4: NK cell input
5: IgG
6: NK cell ANXA6-mAb (IP)

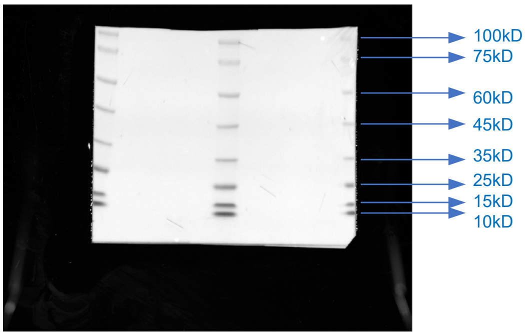

Figure 5G

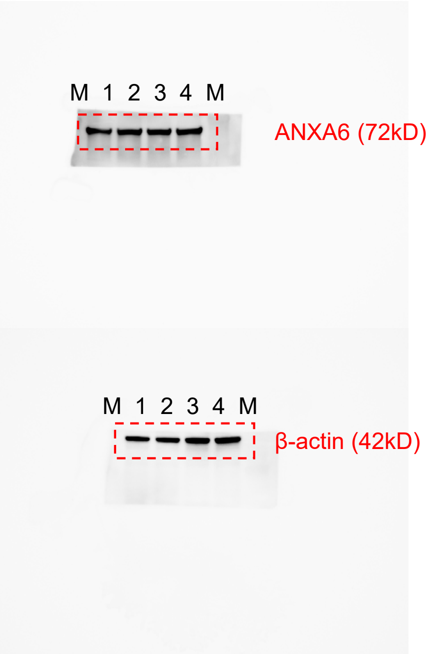

M:180kD protein marker
1: NK cell Control (input)
2: NK cell + CypA (input)
3: NK cell + CsA (input)
4: NK cell + CypA, CsA (input)

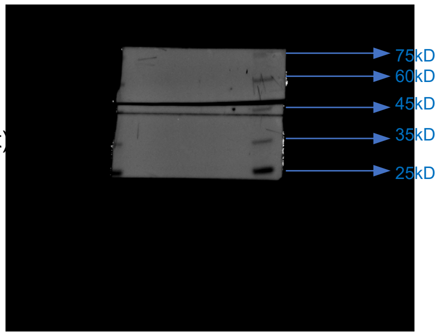

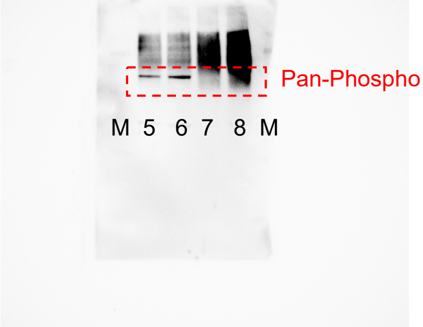

M:180kD protein marker
5: NK cell Control (IP: ANXA6)
6: NK cell + CypA (IP: ANXA6)
7: NK cell + CsA (IP: ANXA6)
8: NK cell + CypA, CsA (IP: ANXA6)

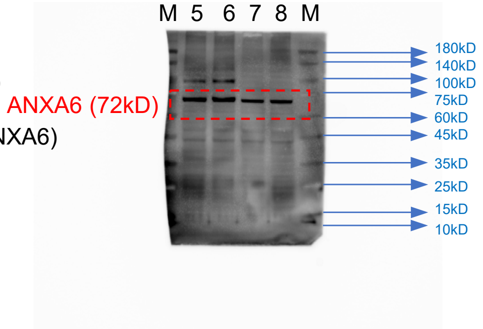

Figure 5H

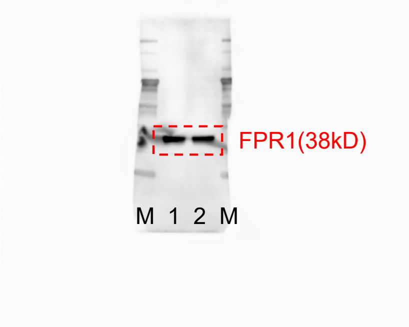

M:180kD protein marker
1: NK cell Control (input)
2: NK cell CypA (input)

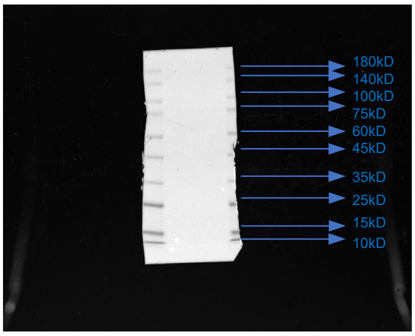

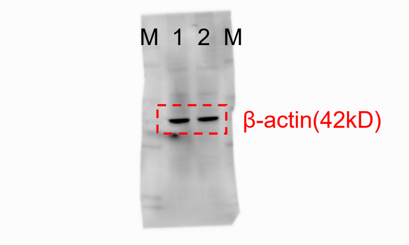

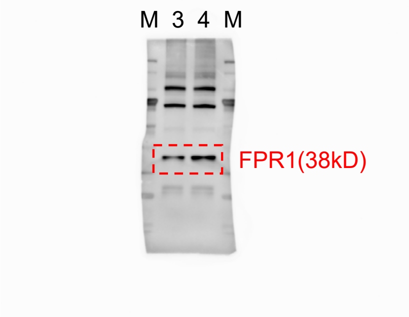

M:180kD protein marker
3: NK cell Control (IP: ANXA6)
4: NK cell CypA (IP: ANXA6)

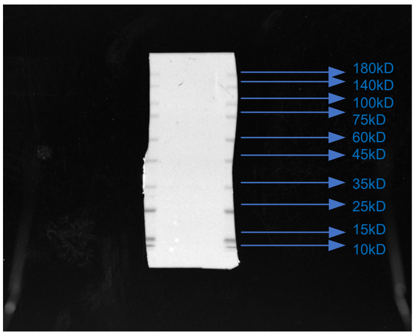

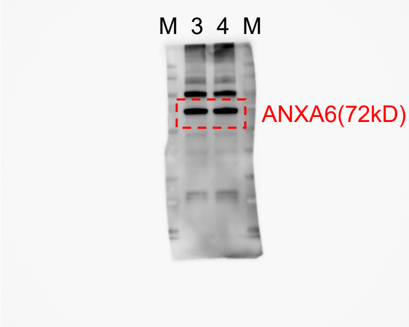

Figure 6E

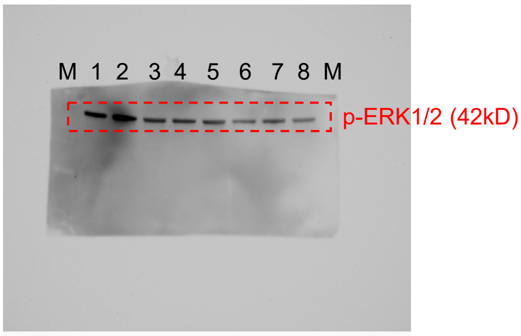

M:180kD protein marker
1: NK cell Control
2: NK cell CypA
3: NK cell CsA
4: NK cell CsH
5: NK cell HCH6-1
6: NK cell CypA, CsA
7: NK cell CypA, CsH
8: NK cell CypA, HCH6-1

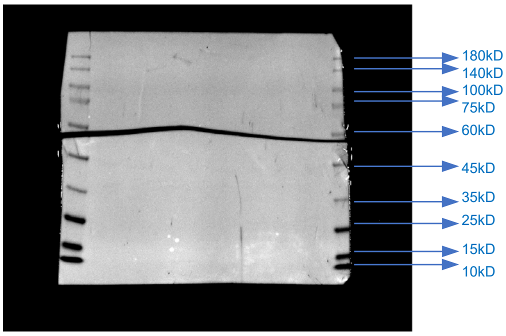

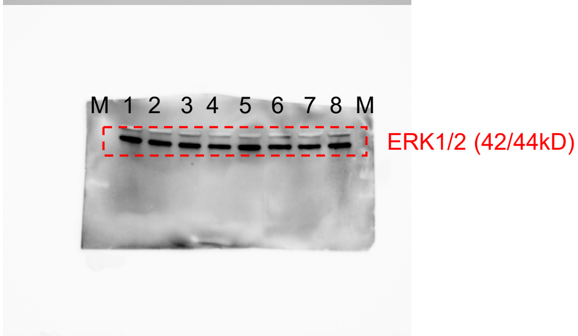

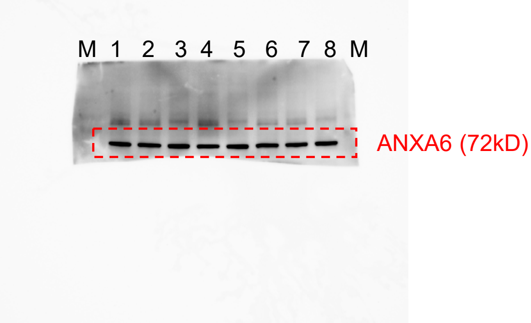

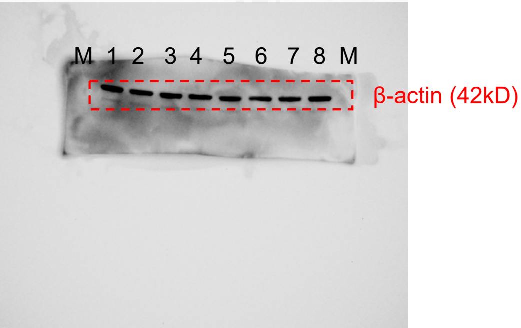

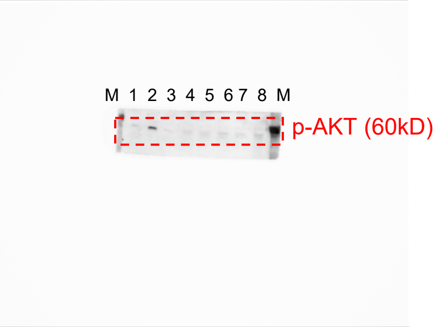

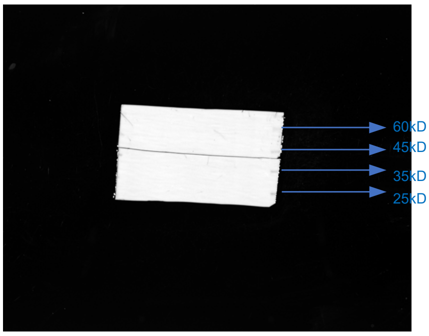

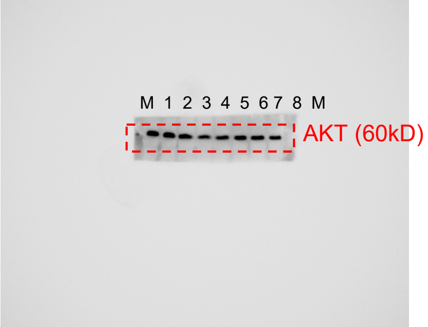

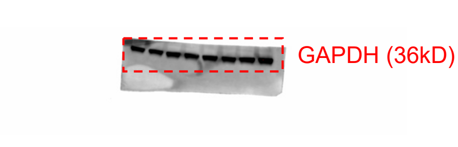

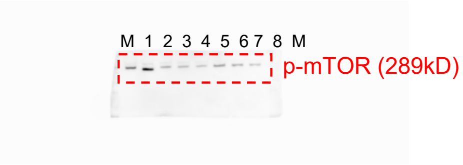

M:310kD protein marker
1: NK cell Control
2: NK cell CypA
3: NK cell CsA
4: NK cell CsH
5: NK cell HCH6-1
6: NK cell CypA, CsA
7: NK cell CypA, CsH
8: NK cell CypA, HCH6-1

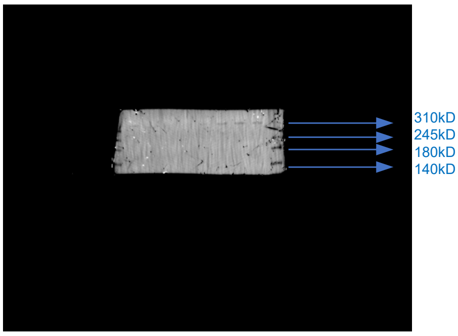

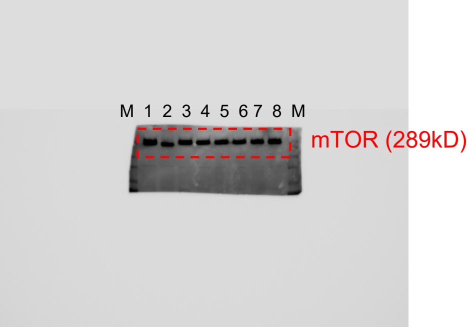

Figure 7J

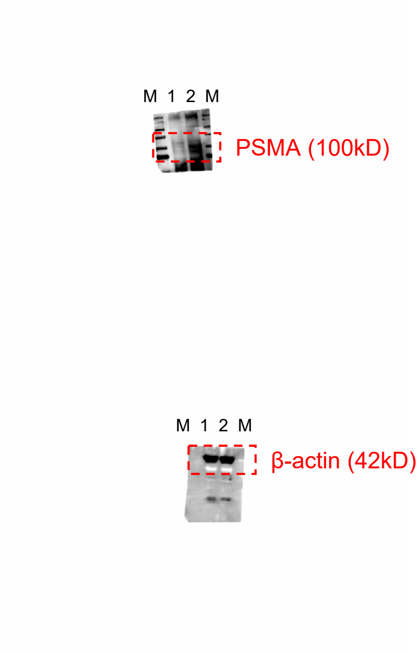

M:180kD protein marker
1: DU145
2: DU145-hPSMA

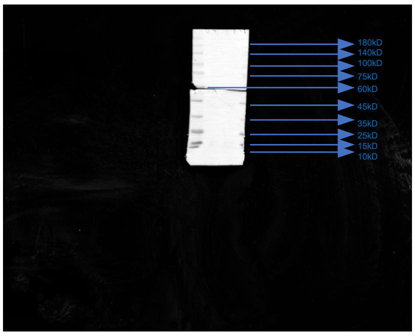

Figure S1D

M:180kD protein marker
1: Patient #1
2: Patient #2
3: Patient #3
4: Patient #4
5: Patient #5
6: Patient #6
7: Patient #7
8: Patient #8
9: Patient #9
10: Patient #10

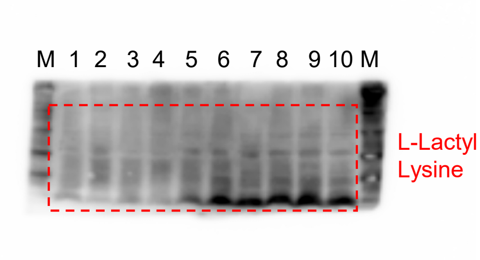

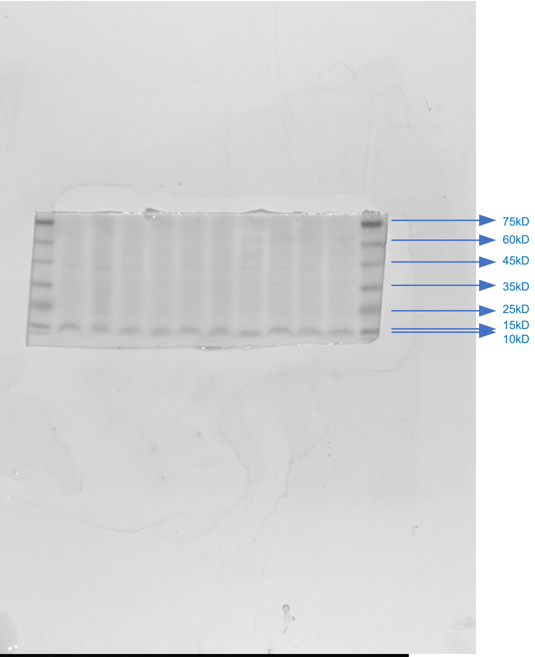

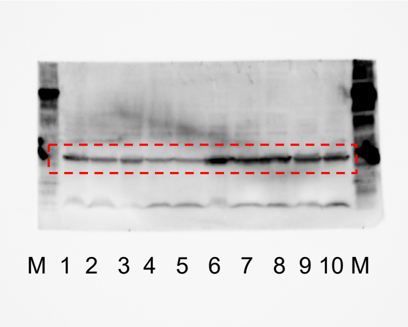

LDHA (37kD)

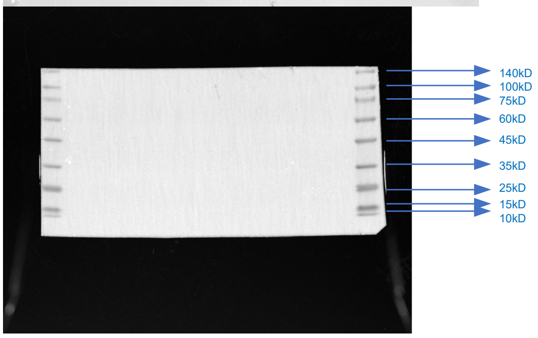

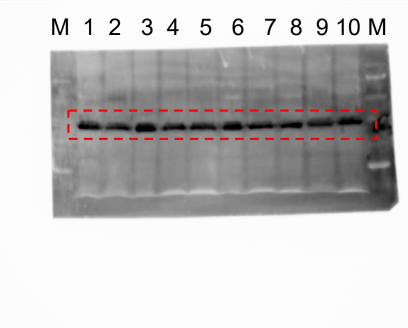

β-actin (42kD)

Figure S2B

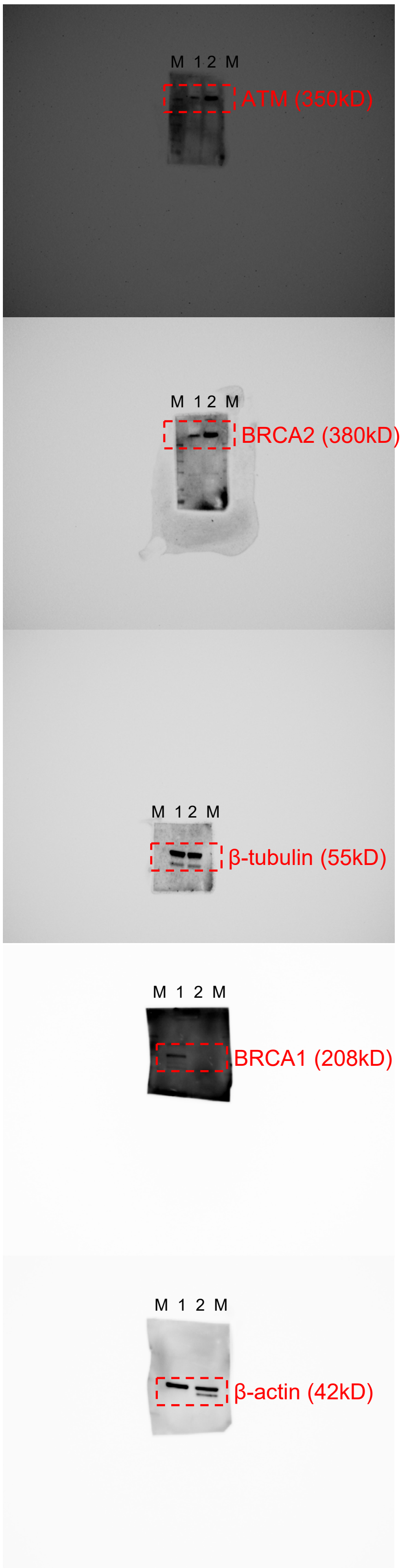

M:310kD protein marker
1: RM-1
2: PPSM

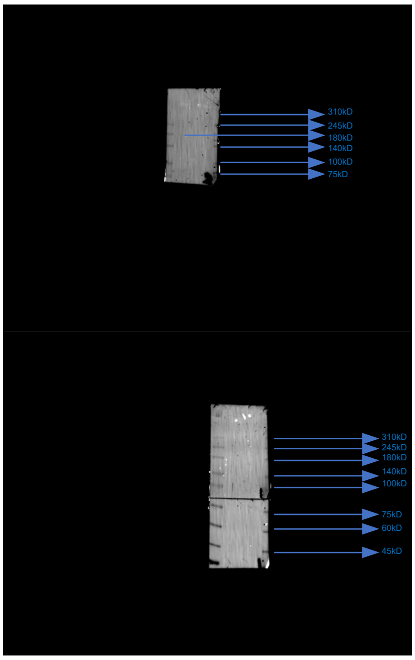

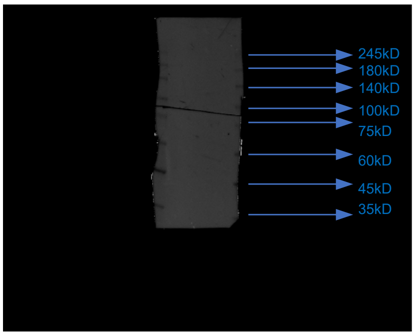

Figure S4D

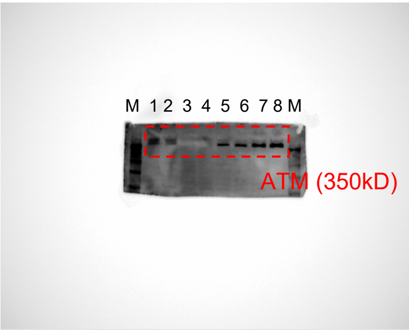

M:310kD protein marker
1: RM-1
2: RM-1
3: Myc-Cap
4: Myc-Cap
5: DU145
6: DU145
7: 22RV1
8: 22RV1

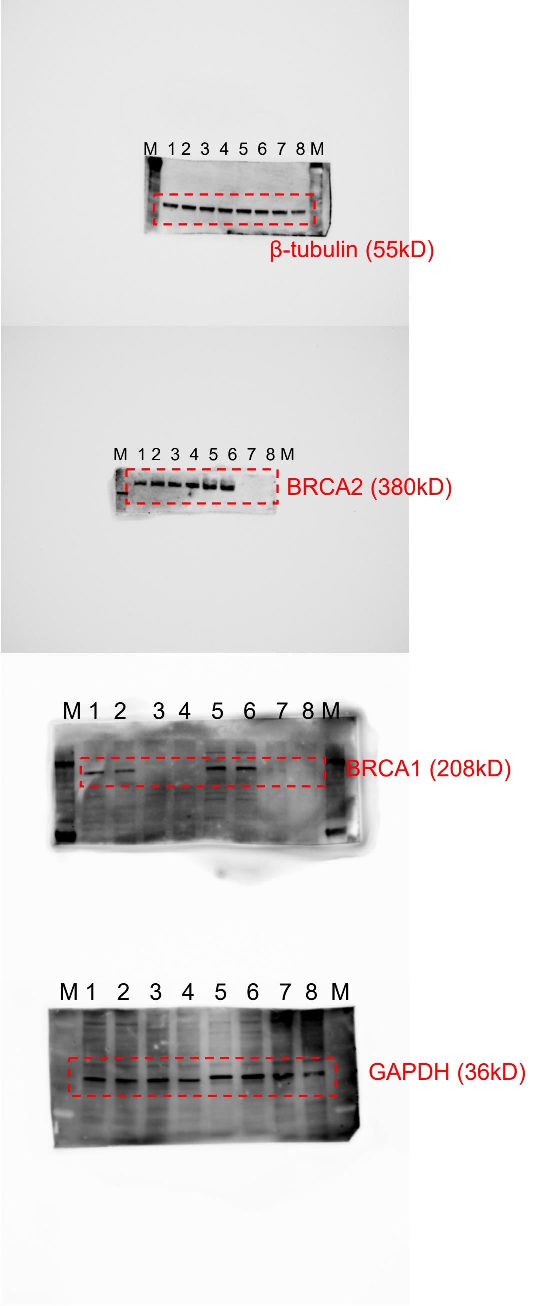

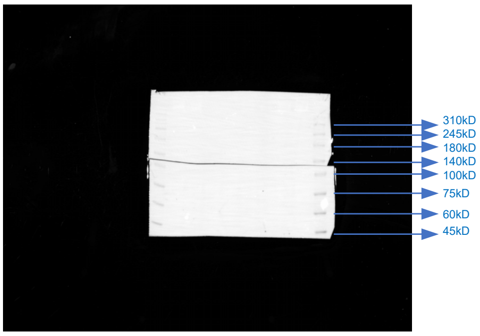

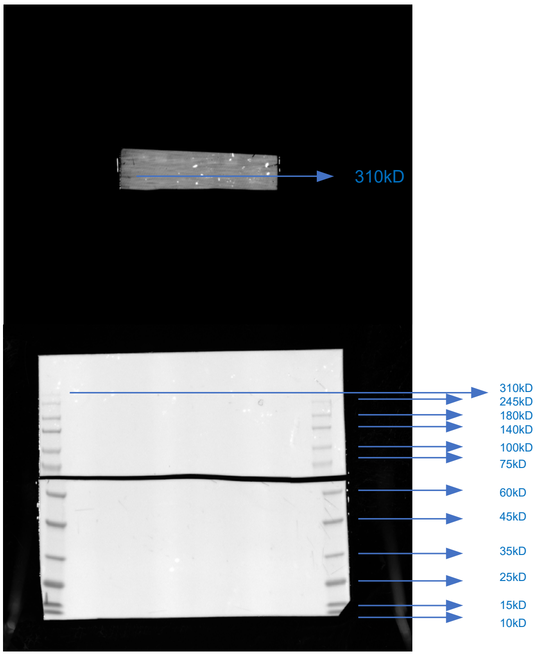

Figure S5D

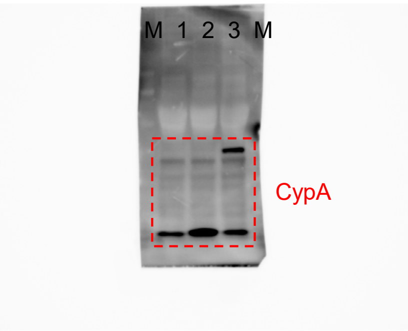

M:180kD protein marker
1: RM-1 Vector
(supernatant)
2: RM-1 CypA overexpression
(supernatant)
3: RM-1 CypA-AcGFP overexpression
(supernatant)

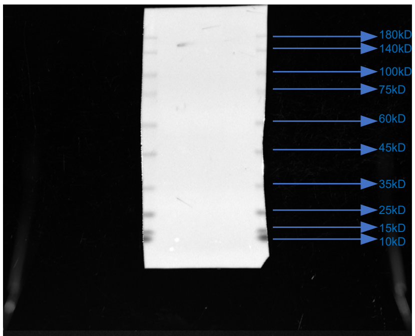

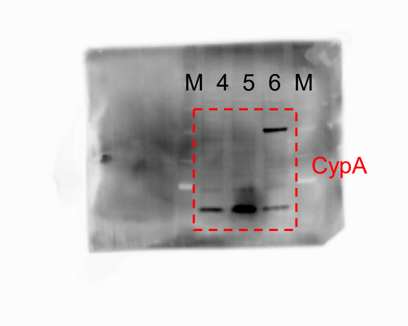

M:180kD protein marker
4: RM-1 Vector
5: RM-1 CypA overexpression
6: RM-1 CypA-AcGFP overexpression

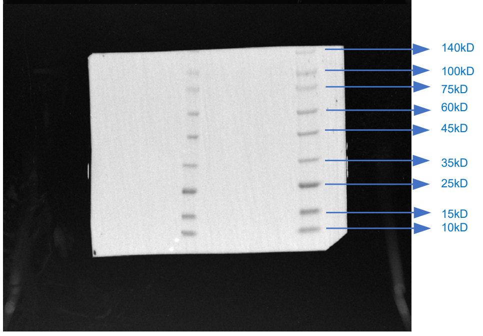

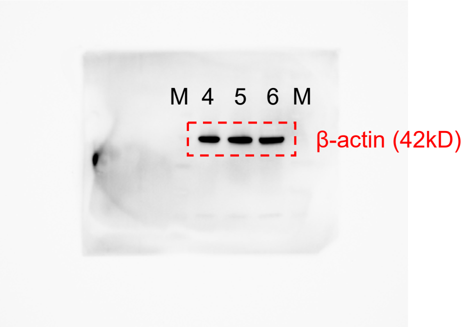

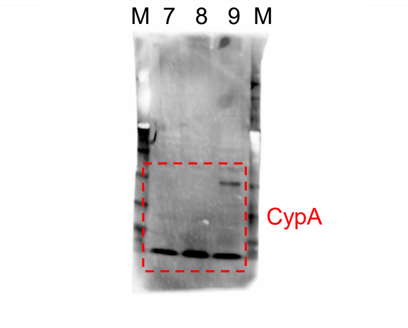

M:180kD protein marker
7: MycCap Vector
(supernatant)
8: MycCap CypA overexpression
(supernatant)
9: MycCap CypA-AcGFP
overexpression
(supernatant)

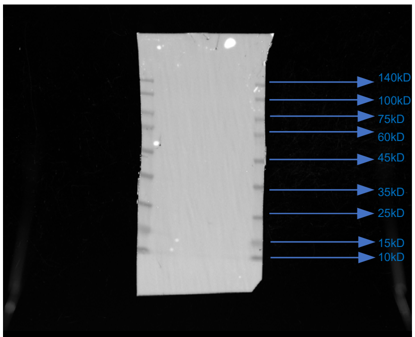

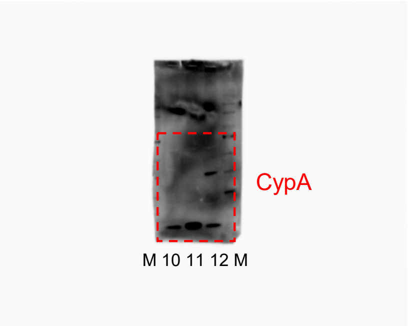

M:180kD protein marker
10: MycCap Vector
11: MycCap CypA overexpression
12: MycCap CypA-AcGFP
overexpression

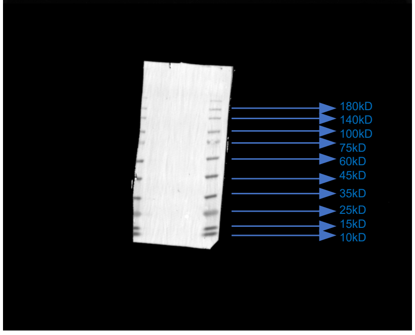

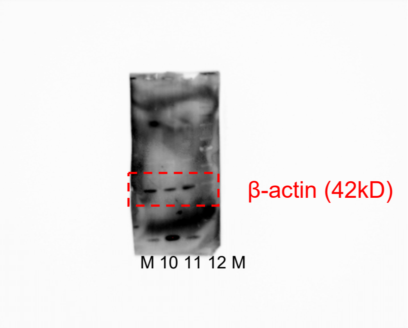

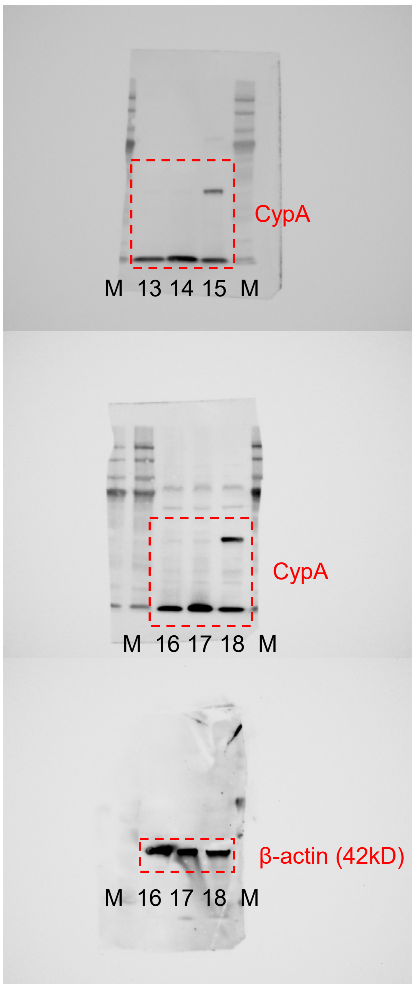

M:180kD protein marker
13: PPSM Vector
(supernatant)
14: PPSM CypA overexpression
(supernatant)
15: PPSM CypA-AcGFP
overexpression
(supernatant)

M:180kD protein marker
16: PPSM Vector
17: PPSM CypA overexpression
18: PPSM CypA-AcGFP
overexpression

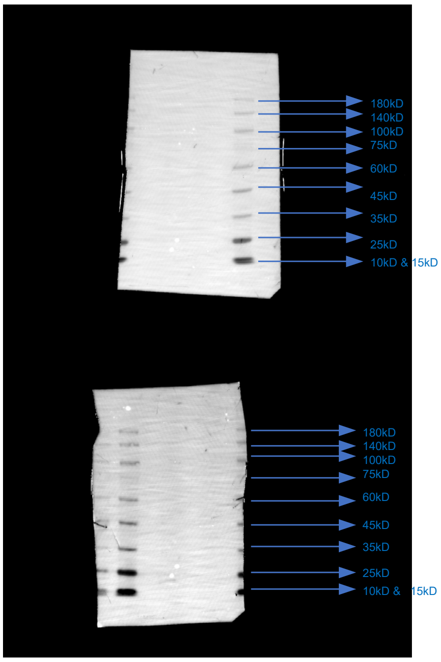

Figure S8A

OT-I
TCR-α
= 350 bp

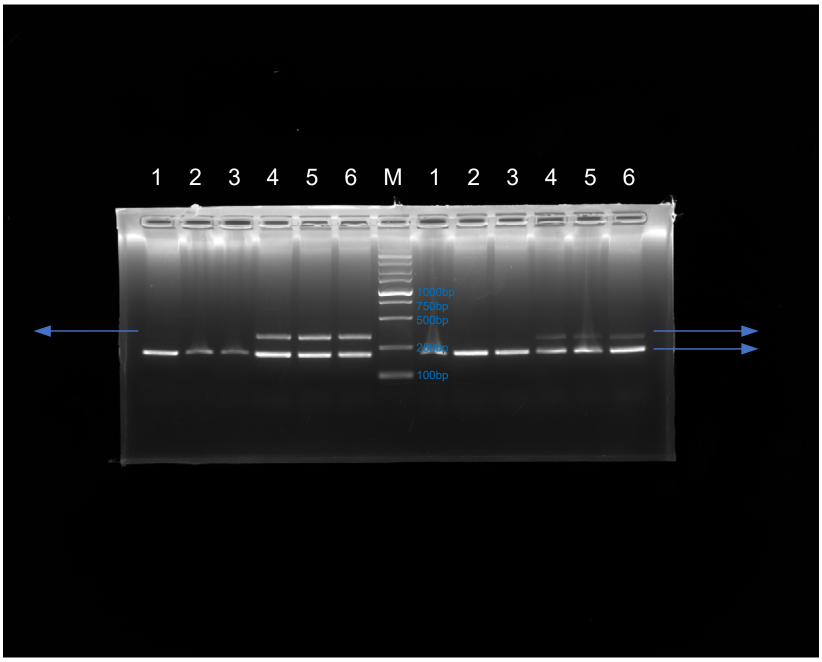

OT-I TCR-β = 300 bp

Internal positive control = 200 bp

M:GN8K DNA Marker
1-3: Wild type C57BL/6
4-6: OT-1 Mice

Figure S10C

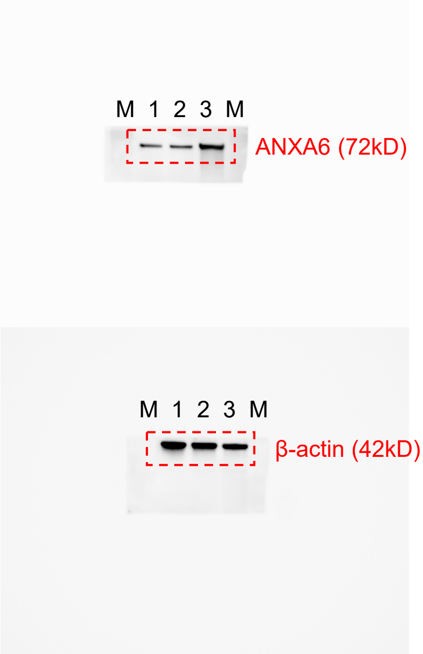

M:180kD protein marker
1: CD4+ T cell (mouse)
2: CD8+ T cell (mouse)
3: NK cell (mouse)

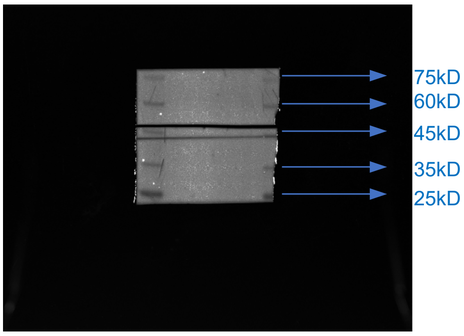

Figure S10D

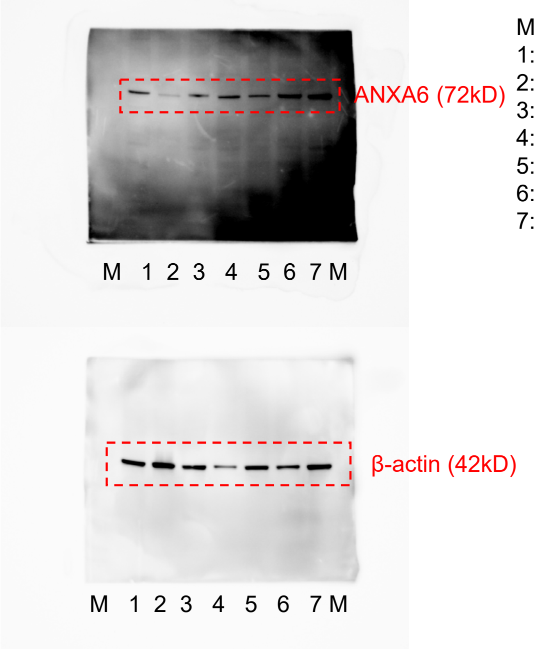

M:180kD protein marker
1: RM-1
2: MycCap
3: PPSM
4: Mb49
5: MC38
6: Renca
7: Hep1-6

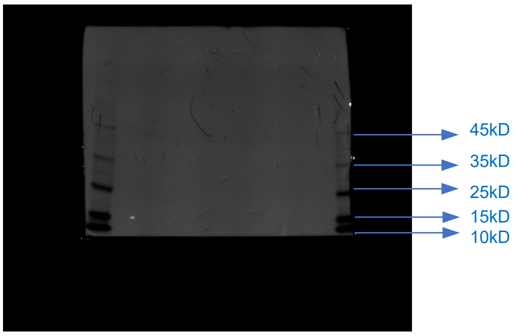

Figure S10E

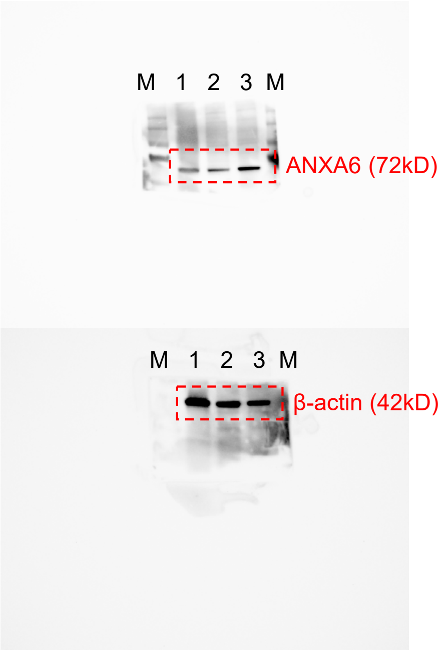

M:180kD protein marker
1: CD4+ T cell (human)
2: CD8+ T cell (human)
3: NK cell (human)

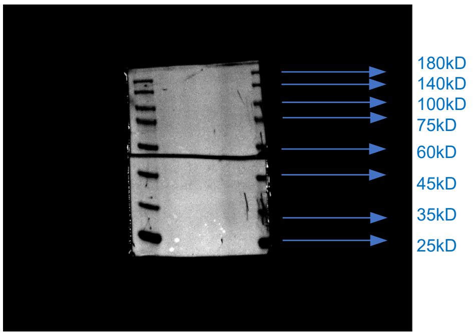

Figure S10F

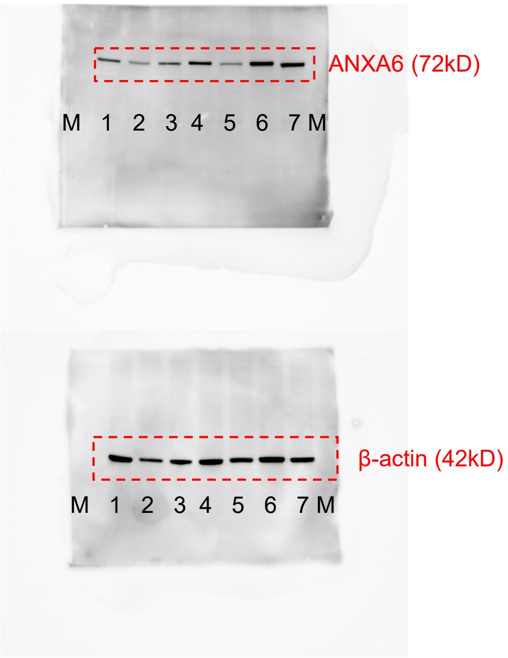

M:180kD protein marker
1: DU145
2: 22RV1
3: PC3
4: T24
5: HCT116
6: 786O
7: HepG2

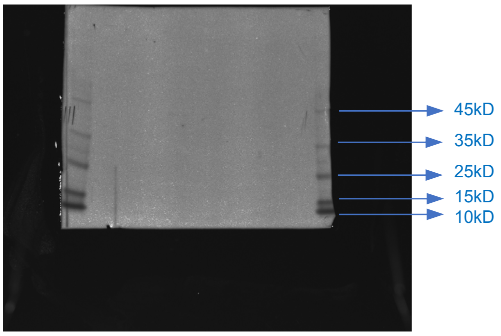

Figure S10H

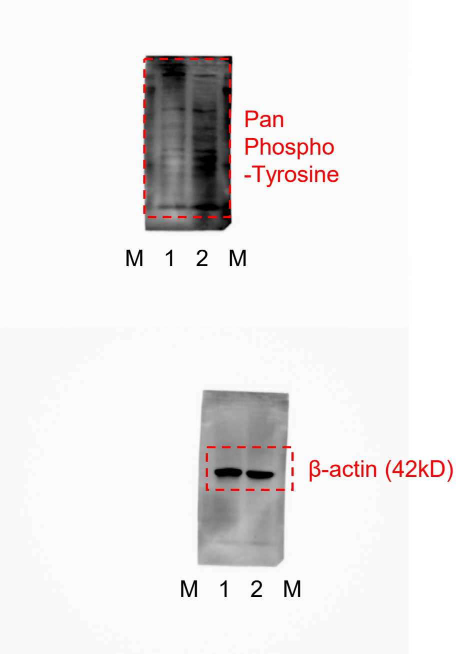

M:180kD protein marker
1: NK cell + BSA
2: NK cell + CypA

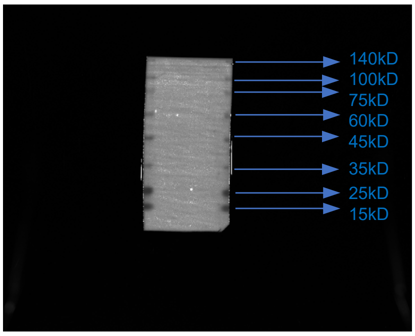

Figure S10I

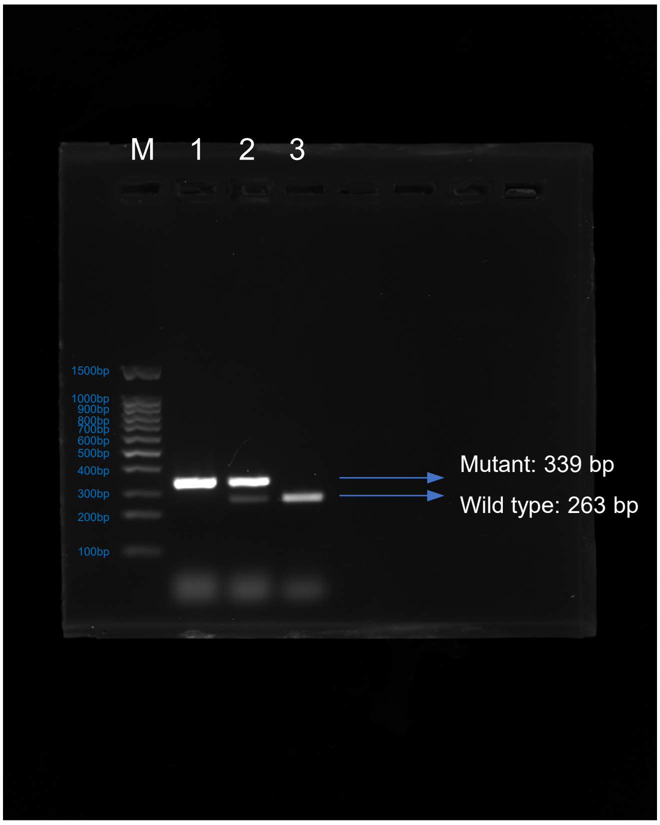

M:100 bp DNA Ladder
1: NCR1-iCre+/+
2: NCR1-iCre+/- (Heterozygote)
3: Wild type C57BL/6

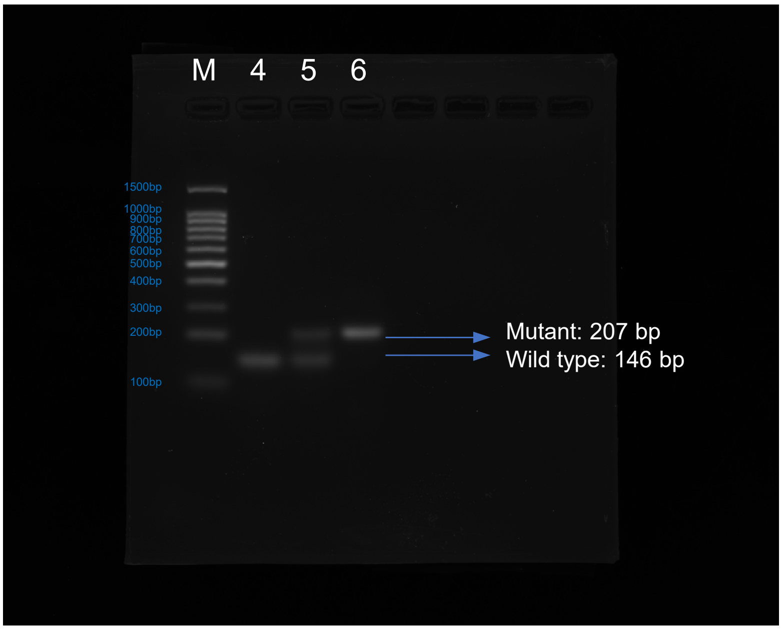

M:100 bp DNA Ladder
4: Wild type C57BL/6
5: FPR1flox/-(Heterozygote)
6: FPR1flox/flox

Figure S10J

M: 180kD protein marker
1: NK cell (WT)
2: NK cell (FPR1 Flox/Flox)
3: NK cell (cKO)

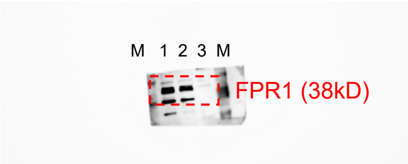

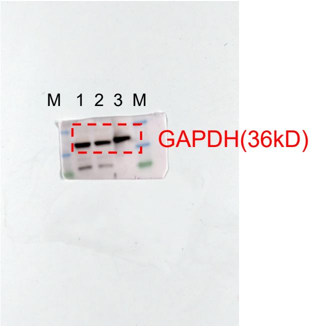

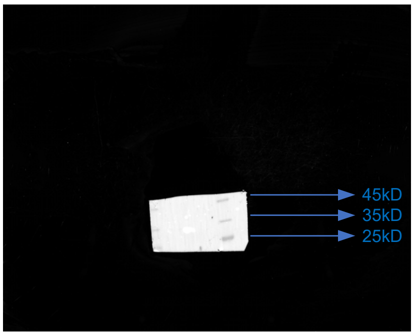

Supplement: Unedited blot and gel images [file jci-136-197157-s313.pdf]
